# An Edible Biopolymeric Microcapsular Wrapping Integrating Lytic Bacteriophage Particles for *Salmonella enterica*: Potential for Integration into Poultry Feed

**DOI:** 10.3390/antibiotics12060988

**Published:** 2023-05-31

**Authors:** Arthur O. Pereira, Nicole M. A. Barros, Bruna R. Guerrero, Stephen C. Emencheta, Denicezar Â. Baldo, José M. Oliveira Jr., Marta M. D. C. Vila, Victor M. Balcão

**Affiliations:** 1 PhageLab—Laboratory of Biofilms and Bacteriophages, University of Sorocaba, Sorocaba 18023-000, SP, Brazil; artopereira@gmail.com (A.O.P.); a.alencastronicole@gmail.com (N.M.A.B.); riberabruna@gmail.com (B.R.G.); stephen.emencheta@unn.edu.ng (S.C.E.); marta.vila@prof.uniso.br (M.M.D.C.V.); 2 Department of Pharmaceutical Microbiology and Biotechnology, University of Nigeria, Nsukka 410001, Enugu, Nigeria; 3 LaFiNAU—Laboratory of Applied Nuclear Physics, University of Sorocaba, Sorocaba 18023-000, SP, Brazil; denicezar.baldo@prof.uniso.br (D.Â.B.); jose.oliveira@prof.uniso.br (J.M.O.J.); 4 Department of Biology and CESAM, University of Aveiro, Campus Universitário de Santiago, P-3810-193 Aveiro, Portugal

**Keywords:** bacteriophage particles, *Salmonella enterica*, edible biopolymeric wrapping, foodborne illness, phage cocktail, antibacterial control

## Abstract

This research work aimed at developing an edible biopolymeric microcapsular wrapping (EBMW) integrating lytic bacteriophage particles for *Salmonella enterica*, with potential application in poultry feed for biocontrol of that pathogen. This pathogen is known as one of the main microorganisms responsible for contamination in the food industry and in foodstuff. The current techniques for decontamination and pathogen control in the food industry can be very expensive, not very selective, and even outdated, such as the use of broad-spectrum antibiotics that end up selecting resistant bacteria. Hence, there is a need for new technologies for pathogen biocontrol. In this context, bacteriophage-based biocontrol appears as a potential alternative. As a cocktail, both phages were able to significantly reduce the bacterial load after 12 h of treatment, at either multiplicity of infection (MOI) 1 and 10, by 84.3% and 87.6%, respectively. Entrapment of the phage virions within the EBMW matrix did not exert any deleterious effect upon their lytic activity. The results obtained showed high promise for integration in poultry feed aiming at controlling *Salmonella enterica*, since the edible biopolymeric microcapsular wrapping integrating lytic bacteriophage particles developed was successful in maintaining lytic phage viability while fully stabilizing the phage particles.

## 1. Introduction

Foodborne diseases are a major cause of morbidity and mortality worldwide. According to the World Health Organization (WHO), it is estimated that diarrheal diseases alone (most of which are caused by foodstuff contaminated by pathogenic microorganisms) kill 1.9 million children per year [1]. Foodborne diseases are a global public health concern and, according to the WHO, it is estimated that one out of ten cases can be fatal, especially in children under five years of age, causing about 420 thousand deaths in the American continent [2]. In Brazil, most foodborne diseases are caused by the pathogens *Salmonella enterica*, *Escherichia coli*, and *Staphylococcus aureus* [3,4], causing diarrhea, abdominal pain, vomiting, and/or nausea [4].

Discovered and described in 1885 by Daniel Salmon, a veterinary bacteriologist, the genus *Salmonella* is considered a member of the Gram-negative *Enterobacteriaceae* family [5]. It is commonly divided into two species, *Salmonella enterica* and *Salmonella bongori*, and has over 2500 known serotypes that differ in their wide host range and ability to cause disease, with over 50% of the identified serotypes belonging to the *Salmonella enterica* species, which is responsible for the vast majority of *Salmonella* infections in humans [6]. *Salmonella* is part of the microbiota of birds, and there is a diverse amount of *Salmonella* serovars. While the serovars *Salmonella pullorum* and *Salmonella gallinarum* are important pathogens of birds, the serovars *Salmonella enteritidis* and *Salmonella typhimurium* have a broad host spectrum [7]. Among other foodborne pathogens, *Salmonella* alone is responsible for several tens of millions of incidents of salmonellosis worldwide, on a yearly basis, associated with more than 150,000 deaths [4,5].

From the second half of the twentieth century, the emergence of bacterial strains resistant to multiple drugs (viz. multi-drug resistant) has been a reality arising from broad, indiscriminate utilization of chemical antibiotics in areas as diverse as (but not limited to) human medicine, animal medicine, food industry, and agriculture [6,8]. In addition, such multi-drug resistant bacteria may be transmitted from direct contact between farmers and animals and the environment [9]. Antibiotics that promote animal growth have been used since the last three quarters of a century with the aim of improving both animal health and performance. Despite this, and due to the appearance of resistance to antibiotics in bacteria associated with public health hazards, routine supplementation of antibiotics in animal production has been drastically reduced and even banned in some countries [10].

Poultry production is one of the world’s sectors that most uses antibiotics, and reducing the use of antibiotics is one of the biggest challenges for this industry globally [9]. In this context, alternative approaches have become necessary, with the application of lytic bacteriophages (or phages) being a potential alternative to combat bacterial diseases in the agricultural industry [11], with enormous potential in the fight to reduce the burden of infectious diseases [2,12].

Bacteriophages are viruses devoid of metabolic machinery of their own that exclusively infect susceptible bacterial cells, hence being obligate intracellular parasites that require a bacterial host cell to replicate [13,14]. The use of bacteriophages to biocontrol bacteria has unique advantages, including that these viral particles are natural, self-multiplying, and highly specific antibacterial agents [13,14]. In addition, bacteriophages specifically target their bacterial host cells while not affecting the local microbiota, are self-replicating and self-limiting nano-entities while there are still viable target host cells, can adapt to the major defense mechanisms of the target host cells, display virtually nil toxicity, are easy and economical to isolate, and can tolerate various conditions prevailing in food matrices [13]. In this way, researchers have sought to use them to treat various types of bacterial infections in humans and animals as well as in environmental applications [5,13,15]. According to several researchers, the concept of combating pathogenic bacteria in food by using phage particles can be addressed at all stages of the entire food chain, specifically in preventing or reducing colonization and disease in livestock via phage therapy [16].

In the poultry industry, bacteriophages have also been used in a wide variety of applications, such as treating live birds, adding to poultry products, and disinfecting processing equipment. It was shown that treatment with phages administered to chickens via aerosol or oral gavage was able to control bacterial infections and decrease mortality [17]. Recent studies have reported success in reducing *Salmonella* spp. by the application of bacteriophages in chickens and products derived from them. Additionally, bacteriophage supplementation has been shown to improve feed efficiency, reduce pathogens in broilers, and improve production and egg quality in laying hens [11].

For the success of antibacterial therapy with phages, several obstacles still have to be overcome, with one of the problems of phage therapy for birds (especially in large commercial aviaries) being the form of administration. In this sense, the incorporation of phage particles into poultry feeds could be an interesting alternative. For this, the phage must be able to survive in the feed and the gastrointestinal tract of the birds. The acidic environment of the stomach can promote the deactivation of phage particles [18]. Abiotic factors such as pH, temperature, and light radiation are parameters known to affect the stability of bacteriophages and their infectious ability [19]. Luminal pH in the gastrointestinal tract of birds ranges from highly acidic in the proventriculus (pH 2.0–5.0) to slightly basic in the small intestine (pH 5.0–7.0). All feeds fed to chickens are thus subjected to gastric pH in the range of 2.0 to 5.0 [20]. In the case of bacteriophages, for their efficient delivery into the gastrointestinal environment and a safe passage through the acidic environment, the protection of phage particles is a very important factor to achieve the desired antibacterial therapeutic effect of bacteriophages. From this perspective, preventive strategies are necessary to protect the phage particles. One such strategy is the microencapsulation of phages [18,21] aiming at their potential incorporation into poultry feed, which was the strategy followed in the research work entertained herein. Essentially, phage encapsulation is a process whereby the phage particles are coated with appropriate biopolymeric materials to segregate them from the surrounding environment, thus protecting the bacteriophages from the aggressive environment of the bird’s gastrointestinal tract, which could reduce their viability or render them inactive [22] before they could exert their antibacterial action.

With all the aforementioned facts in mind, the major goal of the research work entertained herein was to isolate and characterize lytic bacteriophage particles for *Salmonella enterica* and promote their structural and functional stabilization within biopolymeric microcapsular wrappings aiming at potential applications in poultry feed for the biocontrol of *Salmonella enterica* in live poultry and the associated foodstuff thereof (eggs and carcasses).

## 2. Materials and Methods

### 2.1. Biological Material

***Bacterial host for phage isolation***: The collection *Salmonella enterica* CCCD-S004 strain utilized in this work as host for phage isolation was acquired from CEFAR Diagnóstica (São Paulo, SP, Brazil). ***Bacteriophages***: The two phages utilized in this study (ph001L and ph001T) were previously isolated from samples of lake water (ph001L) and soil with hen faeces (ph001T) collected near the Veterinary Hospital at UNISO (geographic coordinates: 23°29′58.7″ S; 47°23′45.2″ W), Sorocaba/SP (Brazil). ***Collection strains for host-range assays***: The bacterial strains utilized in the extended host-range assays were obtained either from ATCC (American Type Culture Collection, Gaithersburg, MD, USA) (viz. *Aeromonas hydrophyla* ATCC 7966, *Salmonella thyphimurium* ATCC 13311, *Escherichia coli* ATCC 8739, *Enterococcus faecalis* ATCC-29212, *Klebsiella pneumoniae* ATCC-13883, *Salmonella enterica* subsp. Enteritidis ATCC 13076, *Proteus mirabilis* ATCC 25933, *Pseudomonas aeruginosa* ATCC 27853, *Pseudomonas aeruginosa* ATCC 9027, *Staphylococcus aureus* ATCC 25923, *Staphylococcus aureus* ATCC 6538, *Salmonella enterica* subsp. *enterica* serovar Typhimurium ATCC 14028, *Bacillus cereus* ATCC 14579, and *Escherichia coli* ATCC 25922), CEFAR (São Paulo, SP, Brazil) (viz. *Pseudomonas aeruginosa* CCCD-P004, *Salmonella enterica* CCCD-S004, and *Proteus mirabilis* CCCD-P001), IBSBF (Phytobacteria Culture Collection of Instituto Biológico Campinas, SP, Brazil) (viz. *Pseudomonas syringae* pv. *garcae* IBSBF-158), and NCTC (National Collection of Type Cultures, UK Health Security Agency (UKHSA), Salisbury, UK) (viz. *Klebsiella pneumoniae* NCTC-13439). All manipulation of materials, bacteria, bacteriophages, culture media, and evaluation of microbiological activity was carried out in a Filterflux^®^ Class II, type B2, biological safety cabinet model SP-SBIIB2-126 from SP-LABOR (Presidente Prudente, SP, Brazil).

### 2.2. Chemicals

The chemicals utilized in this study were purchased from Dinâmica Química Contemporânea Ltd.a (Diadema, SP, Brazil). Tryptic Soy Agar (TSA) and Tryptic Soy Broth (TSB) culture media were purchased from Sigma-Aldrich Brazil (Cotia, SP, Brazil), and bacteriologic solid agar was purchased from Gibco Diagnostics (Madison, WI, USA). Sterilizing filtration systems Stericup™-GP (with 0.22 µm pore diameter polyethersulphate membrane) were acquired from Merck-Millipore (Darmstadt, Germany). Tap water was ultra-purified to a final resistivity of 18.18 MΩ·cm and conductivity of 0.05 µS·cm^−1^ in a Master System All MS2000 (Gehaka, São Paulo, SP, Brazil).

### 2.3. Preparation of a Salmonella enterica CCCD-S004 Growth Curve

The host bacteria (in lyophilized form, from CEFAR collection) was hydrated in TSB liquid medium, plated on solid TSA, and incubated at 37 °C for 12 h. A single CFU was then withdrawn with a sterile loop, inoculated in 250 mL sterile TSB, and incubated at 37 °C for 12 h. At predetermined time intervals, the optical density of the culture was evaluated spectrophotometrically at 610 nm.

### 2.4. Phage Enrichment, Isolation, Propagation, and Enumeration

Phage enrichment from samples of lake water and soil with hen faeces collected near the Veterinary Hospital at UNISO was performed according to the procedure described elsewhere [23,24,25], with small modifications, using *Salmonella enterica* CCCD-S004 (in exponential growth).

Isolation of phage plaques was carried out using the conventional double-layer agar method described in previous works [23,24,25,26], and titres (PFU/mL, plaque-forming units/mL) of the phage suspensions produced thereof were determined.

A *Salmonella enterica* overnight culture (100 µL) was mixed with 5 mL of molten top agar–TSB (30 g/L TSB, 6 g/L agar, 0.05 g/L CaCl_2_, 0.12 g/L MgSO_4_, pH 7.4) in test tubes, tapped gently, and poured onto TSA plates which were gently swirled and allowed to dry out for 1–2 min, followed by overnight incubation at 37 °C. Sterile paper strips were wetted in the phage enrichment suspension and dragged several times on a Petri plate containing a bacterial lawn. The plate was incubated at 37 °C for 24 h and then the morphology of the phage plaques was observed. Different phage plaques were pierced with sterile toothpicks, which were then stuck several times (in a line) in Petri plates with bacterial lawn. Sterile paper strips were then used to streak the phages as described above. More successive single-plaque isolation cycles were performed to obtain pure phage isolates. The plates with different plaque morphologies were then incubated overnight at 37 °C, and the last two steps were repeated until all phage plaques were uniform. The plates were stored at 4 °C until needed. To each plate used, 5 mL of SM buffer was added, and the plates were then further incubated with shaking (70 rpm) at 4 °C for 18 h. After incubation, the SM buffer with phages was collected and added to chloroform up to a final ratio of 10% (*v*/*v*). The phage suspensions were centrifuged (9000× *g*, 4 °C, 10 min) to remove intact bacteria or bacterial debris. The aqueous phases were collected into a single sterile flask and stored at 4 °C as phage stocks.

Bacteriophage enumeration was carried out via the double agar overlay technique, as follows. Serial dilutions of the concentrated stock bacteriophage suspensions produced were prepared sequentially, starting by adding 50 µL of each concentrated stock bacteriophage suspension to 450 µL SM buffer. An amount of 5 µL-droplets of each bacteriophage dilution were plated in triplicate in a lawn of the bacterial host and the plates were allowed to dry out for 10 min, after which they were incubated overnight at 37 °C. Following incubation, the bacteriophage plaques formed in each serial dilution were counted, considering only those dilutions with 3–30 bacteriophage plaques. The bacteriophage titre (PFU/mL) of the concentrated stock bacteriophage suspensions was then calculated as number of phage plaques formed×1dilution×1Vbacteriophage inoculum mL.

### 2.5. Phage–PEG Precipitation

Phage suspensions (10^10^–10^11^ PFU/mL) were added to a sterile mixture of polyethylene glycol (PEG) 8000 (Sigma-Aldrich, St. Louis, MO, USA) (10%, *w*/*w*) and NaCl (1 M) (Sigma-Aldrich, St. Louis, MO, USA), in a volumetric proportion of 2:1, respectively. The resulting suspensions were incubated overnight at 4 °C and then centrifuged at 11,000 rpm (4 °C, 45 min). The supernatant was then discarded, and the pellet was resuspended and homogenized in a 5 mM MgSO_4_ aqueous solution (Sigma-Aldrich, St. Louis, MO, USA).

### 2.6. UV-Vis Spectral Scans for Determination of Phage Particle Extinction Coefficient

Determination of the phage particle molar extinction coefficient was based on the procedure described elsewhere [23,24,25,26,27], using the wavelengths producing the maximum absorption of phage particles (viz. 252 nm (phage ph001L) or 251 nm (phage ph001T)) and 320 nm (wavelength where phage chromophores produce little light absorption). All spectrophotometric readings were performed in a UV-Vis spectrophotometer from Agilent (model Cary 60 UV-Vis, Santa Clara, CA, USA).

### 2.7. Transmission Electron Microscopy (TEM) Analyses

Phage particles of PEG-concentrated suspensions were centrifuged (4 °C, 150 min, 45,000 rpm, 124,740× *g*) in a benchtop Beckman-Coulter ultracentrifuge (model Optima TLX micro-ultracentrifuge) with a TLA-55 Fixed-Angle Rotor (Indianapolis, IN, USA) and underwent negative staining with uranyl acetate (Sigma-Aldrich, St. Louis, MO, USA) at 2% (*w*/*v*) and pH 7.0, following the procedure described in previous works [23,24], prior to analysis via transmission electron microscopy in a transmission electron microscope from JEOL (model JEM 2100, Tokyo, Japan), encompassing an LaB_6_ filament, operating at 200 kV and with resolution of 0.23 nm; a high-resolution CCD camera from GATAN Inc. (model ORIUS™ 832.J4850 SC1000B, Pleasanton, CA, USA) with a resolution of 11 Mp (4.0 × 2.7 k·pixels/9 × 9 µm^2^) was utilized for the acquisition of digital images, via the software Gatan Microscopy Suite (DigitalMicrograph from Gatan Inc., version 2.11.1404.0, Pleasanton, CA, USA).

To determine virion capsid and tail dimensions, 7 phage particles were measured for each phage using the public domain ImageJ software (version 1.52a) from the National Institute of Health (Bethesda, MD, USA).

### 2.8. Host Range of Isolated Phage Particles: Spot Test and Efficiency of Plating (EOP)

Phage host-range was determined by spot-testing using the bacterial strains listed in Table 4, according to the procedure described elsewhere [25,28,29]. For those bacterial strains that produced positive spot tests, the EOP was calculated by comparison with the efficacy for *Salmonella enterica* CCCD-S004 (isolation host, EOP = 100%), as $\mathrm{EOP}=\left(Average\;PFU_{target\;bacteria}\;/Average\;PFU_{host\;bacteria}\right)\times 100$ [23,24,25,28,30,31]. All EOP data displayed in Table 4 represent averages of three separate experiments, and were scored as high (≥50%), moderate (10–0.1%), low (0.1–0.001%), or inefficient (≤0.001%), relative to the isolation host (100%) [31].

### 2.9. Phage One-Step Growth (OSG) Analyses

Growth parameters for the two phages were extracted from the one-step growth curves using *Salmonella enterica* CCCD-S004 (1 × 10^8^ CFU/mL) and ph001L (1 × 10^5^ PFU/mL) and ph001T (1 × 10^5^ PFU/mL) at MOI ≤ 0.001 [23,25], with three independent experiments. Adjusting a typical sigmoidal model such as a four-parameter logistic (4-PL) regression equation, viz. $Log\;\left(P_{t}\right)=P_{\infty }+\left\{\left(P_{0}-P_{\infty }\right)/\left(1+{\left(\frac{t}{\psi }\right)}^{\xi }\right)\right\}$, to the experimental phage growth data was a natural sequence. This allowed one to determine phage growth characteristics such as eclipse, latent and intracellular accumulation periods, and virion progeny yield [23,24,25,29]. In the model just described, *P_t_*, *P*_0_, and *P*_∞_ are phage concentrations (PFU/mL) at times *t*, 0, and ∞, respectively, ψ is the curve inflection point, ξ is the curve steepness (Hill’s slope), and *t* is the incubation time (min). The model was fitted to the experimental phage growth data via nonlinear regression analysis using the function “Solver” within Microsoft Excel (Microsoft, Redmond, WA, USA).

### 2.10. Phage Adsorption Analyses

A *Salmonella enterica* CCCD-S004 suspension in exponential growth (OD_610nm_ ≈ 0.5, ≈ 5 × 10^8^ CFU/mL) was added to bacteriophage particles at 8 × 10^5^ CFU/mL in order to produce MOI 0.001 [32], so that phage particles had a bacterial cell to adsorb onto, and the determinations proceeded in three independent assays as previously described [23,24,25]. Phage particle adsorption onto the host cells was expressed as normalized phage concentration in the supernatant along incubation time. If one assumes that phage virions have the ability to adsorb onto susceptible cells and establish a reversible complex involving both bacterial cells and adsorbed virions leading either to an infected bacterium or to a non-infected counterpart, according to the mechanistic representation deployed by several researchers [33,34,35], viz. $\mathrm{Free}\;\mathrm{virion}\;\left(P\right)+\mathrm{bacterium}\;\left(X_{0}\right)\;\begin{array}{c} \delta \cdot X_{0}\\ ⇄\\ \phi \end{array}\;\mathrm{Reversible}\;\mathrm{complex}\;\left\{\mathrm{virion}-\mathrm{bacterium}\right\}\;\left(\Delta \right)$, resulting in postulation of the mathematical model $\frac{P_{t}}{P_{0}}=\frac{\phi +\left\{\delta \;\times X_{0}\times {\;e}^{-\left(\delta \;\times X_{0}+\phi \right)\times t\;\;}\right\}}{\delta \;\times X_{0}+\phi }$, where *P_t_* and *P*_0_ are phage concentrations (PFU·mL^−1^) at times *t* and 0, respectively, δ is the (first order) phage virion adsorption rate onto susceptible bacteria (CFU^−1^·mL·min^−1^), ϕ is the (first order) phage virion desorption rate from reversible virion–bacteria complexes (mL·min^−1^), *X_0_* is the initial concentration of uninfected (but susceptible) bacteria (CFU/mL), and *t* is infection time (h). The model was then fitted to the experimental phage adsorption data via nonlinear regression analysis using the function “Solver” within Microsoft Excel (Microsoft, Redmond, WA, USA), allowing determination of the phage adsorption rate.

### 2.11. Bacteria Inactivation Experiments In Vitro by the Two Phages

Inactivation of planktonic host cells (10^5^ CFU/mL, exponential growth) by the phages was studied at MOI values 0.01, 0.1, 1, 10, 100, and 1000. For each MOI experiment (performed in triplicate in three independent assays), a bacterial control (BC) was also added, comprising only planktonic host cells. Both BC and treatments (BP-B, bacteria, and phage–bacterial concentration) were incubated with the same time/temperature parameters, viz. 37 °C and 12 h. Two mL-aliquots of BC and treatment samples (BP-B) were withdrawn at predetermined intervals of time up to a total treatment timeframe of 12 h, viz. 0, 15, 30, 45, 60, 90, 120, 150, 180, 210, 240, 270, 300, 330, 360, 420, 480, 540, 600, 660, and 720 min, and their absorbance duly measured at a wavelength of 610 nm.

### 2.12. Assessment of the Outcome of Abiotic Factors upon Phage Viability

The aftermath of T, pH, and solar radiation on phage (ph001L and ph001T, 10^7–8^ PFU/mL) viability was studied in phosphate-buffered saline (PBS, 10 mL). For the pH and T experiments, aliquots were withdrawn every 2 h during the first 12 h and then every 12 h up to 72 h of incubation. For the solar radiation experiments, aliquots were withdrawn every 1 h up to 7 h of exposure to direct sunlight. Phage concentrations were evaluated in triplicate in three independent experiments through double-layer agar plating followed by overnight incubation at 37 °C.

#### 2.12.1. pH Studies

The aftermath of pH on phages ph001L and ph001T viability was studied via addition of phage suspensions to sterile PBS with different pH values (viz. 3.0, 6.5, 8.0, 9.0, 10.0, and 12.0), under constant temperature (25 °C).

#### 2.12.2. Temperature Studies

The aftermath of T on phages ph001L and ph001T viability was studied via addition of phage suspensions to sterile PBS (pH 7.0), followed by incubation at a constant temperature (25, 41, and 50 °C).

#### 2.12.3. Solar Radiation Studies

The aftermath of solar radiation on phages ph001L and ph001T viability was studied via addition of phage suspensions to sterile PBS (pH 7.0), followed by exposure to natural sunlight (these were the test samples, S). Control samples (SR-C) were not exposed to solar radiation. These studies were carried out on a shiny day with ambient T varying from 29 °C (09h00) to 35 °C (end of the experiment), with a solar irradiance of ca. 4.561 kWh/m^2^ (data obtained from https://globalsolaratlas.info/map?c=-23.499789,-47.400936,11&s=-23.499789,-47.400936&m=site (accessed on 3 April 2023)). Solar radiation (specifically, UV irradiation) is the most important factor for the loss of phage infectivity in the environment [28]. Short radiation wavelengths (UV-B, 290–320 nm) impart irreversible damages to the phage virion genome and result in modification of viral proteins and formation of (lethal) photoproducts [28]. In the experiments performed herein, one used small transparent flasks made of ordinary (non-mineral) glass, so that UV-A and UV-B radiation from sunlight could pass through and hit the phage suspensions. At the same time, the opening of the flasks were maintained oriented towards the sun, in such a position that sunlight directly hit the surface of the phage suspensions.

### 2.13. Formulation of the Edible Biopolymeric Microcapsular Wrapping (EBMW) Integrating the Bacteriophage Cocktail

Preparation of the EBMW formulation integrating (or not) the phage cocktail followed the internal gelation procedure described elsewhere [36,37,38], with modifications, aimed at structurally and functionally stabilizing the phage particles. As the source of calcium ions, one used calcium chloride. The phage MOIs used in the formulation of the EBMWs were defined considering an initial bacterial contamination “load” of 1.0 × 10^7^ CFU·mL^−1^. Hence, the process (Table 1) was initiated via the preparation of sodium alginate dispersions in ca. 80% (*w*/*w*) of the total mass of ultrapure water containing the phage cocktail. Occasionally, these dispersions were stirred and allowed to stand by for at least 1 h, to allow complete hydration of the sodium alginate. For the EBMW formulation, 5.0 mL gelatin solution at a concentration of 10 mg/mL and 5 mL of the phage cocktail at a given MOI were thoroughly mixed together in a beaker and, afterwards, the resulting suspension was added to 20 mL of sodium alginate at 2% (*w*/*w*). The suspension thus produced (dispersion A, Table 1) was then dripped on 10 mL of a 2.65 mol·dm^−3^ calcium chloride solution containing chitosan at 0.3% (*w*/*w*) (dispersion B, Table 1). After the formation of the EBMWs, the pH was measured and adjusted to 5.5, and the EBMW particles were stored at 4 °C.

### 2.14. Assessment of the Lytic Viability of Entrapped Bacteriophage Particles within the EBMW Formulations

To check the preservation of lytic viability of the bacteriophage particles entrapped within EBMW, a sample of the formulation was placed on a bacterial lawn of the host bacteria on a Petri plate followed by incubation at 37 °C for 24 h. After this time period, the presence (or not) of clear lysis zones surrounding the EBMW sample was observed.

### 2.15. Bacteria Inactivation Experiments In Vitro by the EBMW Particles with Entrapped Phage Cocktail

Inactivation of planktonic *(Salmonella enterica)* host cells (OD_610nm_ ≈ 0.5, exponential growth) by the EBMW particles with entrapped phage cocktail at MOI values 100 and 1000 was studied. For each EBMW formulation (performed in triplicate in three independent assays), a bacterial control (BC) was also prepared, comprising only planktonic host cells. Both BC and treatments (EBMW-BP-B, bacteria, and EBMW formulation—bacterial concentration) were incubated with the same time/temperature parameters, viz. 37 °C and 12 h. A total of 10 microparticles of a given formulation were placed in a 0.5 mL conical-bottom bioreactor containing a Teflon-coated conical stirring bar. An amount of 500 microlitres of bacterial suspension (OD_610nm_ ≈ 0.5, exponential growth) was then added, magnetic stirring (75 rpm) was initiated, and the bioreactor was placed in an incubation chamber set at 37 °C. Five μL-aliquots of BC and treatment samples (EBMW-BP-B) were withdrawn at predetermined intervals of time up to a total treatment timeframe of 12 h, viz. 0, 15, 30, 45, 60, 120, 240, 360, 480, and 720 min, and serial diluted in 45 μL SM buffer. The bacterial concentration in both BC and EBMW-BP-B dilutions was determined in triplicate in solid TSA medium via the drop (5 μL)-plate method after an incubation period of 12 h at 37 °C.

### 2.16. Phage Release Experiments from the EBMW Particles with Entrapped Phage Cocktail

To verify the preservation of lytic activity of the entrapped phage particles and to assess their release from the EBMW particles, a simple experiment was performed. A total of 10 EBMW microparticles with entrapped phages at MOI 1000 were placed in a 0.5 mL conical-bottom bioreactor containing a Teflon-coated conical stirring bar. An amount of 500 microlitres of SM buffer was then added, magnetic stirring (75 rpm) was initiated, and the bioreactor was left at room temperature (ca. 25 °C). Five µL-aliquots of the supernatant were withdrawn at predetermined intervals of time up to a total timeframe of 3 h, viz. 0, 5, 10, 15, 20, 25, 30, 45, 60, 75, 90, and 120 min, and serial diluted in 45 µL SM buffer. The phage titre in all dilutions was determined in triplicate by the double agar-layer method [23,24,25], using 5 µL droplets plated in triplicate in *Salmonella enterica* bacterial lawn (exponential growth phase, OD_610nm_ ≈ 0.5), after an incubation period of 12 h at 37 °C.

### 2.17. Physicochemical Characterization of EBMW Formulation

The physicochemical characterization of the EBMW formulations (Table 1) involved a wide array of analyses (Fourier transform infrared spectrophotometry (FTIR), differential scanning calorimetry (DSC), energy-dispersive X-ray fluorescence (EDXRF), X-ray tomography (XRT), and scanning electron microscopy (SEM)), which will be detailed next.

#### 2.17.1. FTIR Tests

FTIR spectra of EBMW formulations 1 and 5 were obtained in a FTIR spectrophotometer from Thermo Scientific (model Nicolet 6700, Madison, WI, USA) coupled with an ATR module (germanium crystal) (Smart Omni Sampler), from 4000 cm^−1^ to 675 cm^−1^ (resolution: 4 cm^−1^, 1024 scans), with Happ–Genzel apodization.

#### 2.17.2. Thermal Analyses via DSC Tests

Thermal analyses (DSC) of EBMW formulations (16.6180 mg of plain EBMW, and 16.0130 mg of EBMW with phage cocktail at MOI 1000) were performed in a microcalorimeter from METTLER TOLEDO (model DSC-1, Schwerzenbach, Switzerland), according to Rocha et al. [39], using high-pressure aluminum pans sealed by pressure (with the lid punctured with a small hole) containing the samples and a reference aluminum pan with plain air sealed inside. Samples were heated from ca. 25 °C up to 250 °C, at 10 °C·min^−1^, under a constant N_2_ flow of 50 mL·min^−1^, with the heat absorbed by the samples being recorded at a sampling rate of 0.2 s per data point.

#### 2.17.3. EDXRF Tests

The elemental makeup of EBMW formulations (plain and loaded with bacteriophage cocktail at MOI 1000) was determined using an EDXRF spectrometer (model Epsilon 1, Malvern Panalytical, Cambridge, UK) equipped with a 5 W, 10–50 kV, Ag anode X-ray tube, with energy resolution of 125 eV, filters of Ag, Cu, Ti, and Al for the X-ray beams, and a high-resolution 25 mm^2^ silicon drift detector (SDD) operating at P_atm_. All tests were performed with a measuring timeframe of 300 s using atmospheric air, and the spectra were obtained sequentially from 0 keV to 30 keV (resolution of 0.02 keV).

#### 2.17.4. XRT Tests

Tomographic images of the EBMW formulation entrapping the phage cocktail at MOI 1000 were obtained in an X-ray transmission tomograph [40] from Bruker microCT (model SkyScan 1174, Kontich, Belgium). The sample was placed on top of a metallic support coated with adhesive tape which was then placed inside the tomograph chamber. Image slices of the sample were then collected at an operating voltage of 31 kV and electric current of 661 μA. A high number of radiographs (image slices) of the sample were collected via measurement of the X-ray intensities transmitted through the sample at different angular positions (rotation of 180° with angular increments of 0.7° originating 217 radiographs per image (exposure time per radiograph of 2500 ms), each of which holding 1024 × 1304 (width × height) pixels with a spatial resolution of 6.70 μm), so that a tomographic image could be produced. Mathematical algorithms were then utilized to reconstruct the three-dimensional (3D) tomographic images (652 × 652 × 652 pixels) of the EBMW particle, via composition of the bi-dimensional (2D) images acquired. With all the radiographs collected at each angular position, the software NRecon™ (Bruker, version 1.6.9.4, Kontich, Belgium) (using the Feldkamp et al. [41] algorithm to reconstruct the tomographic images), CTVox™ (Bruker, version 2.6.0 r908-64bit), CTan™ (Bruker, version 1.13.5.1-64bit), and CTvol™ (Bruker, version 2.2.3.0-64bit) were utilized for processing all the digital radiographs (image slices).

#### 2.17.5. SEM Tests

The surface and morphology of an EBMW particle were analyzed in a SEM (JEOL, model JSM-IT200, Tokyo, Japan) at high-vacuum. The samples were sputter-coated with a 92 Å-thick Au film via cathodic pulverization, in a metalizing device (JEOL, Sputter Coater model DII-29010SCTR Smart Coater, Tokyo, Japan). Photomicrographs were collected via random scanning using electron beams at acceleration speeds of 10.0 keV.

### 2.18. Statistical Tests

Statistical tests of lack of fit of the mathematical model for phage adsorption (i.e., the expectation function) to the experimental phage adsorption data were undertaken, aiming at testing the goodness of the nonlinear fittings. These statistical tests are based on the fact that the subspace containing the experimental data replications is orthogonal to the subspace containing both the experimental data averages and the expectation function [23,24,28,29,42,43,44]. For this, the *F*-ratio (lack of fit mean square (SS_lack of fit_/NDF_lack of fit_) over the replications mean square (SS_replications_/NDF_replications_)) was compared with the statistical *F*-value (F(ν_NDF, lack of fit_; ν_NDF, replications_; α = 5%)). SS = sum of squares, NDF = number of degrees of freedom.

The data gathered in the in vitro phage–bacteria inactivation assays was statistically analyzed with GraphPad Prism 7.04 (GraphPad Software, San Diego, CA, USA). While the normal distribution of the experimental inactivation data was verified by a Kolmogorov–Smirnov test, the homoscedasticity was verified by the Levene’s test. The significance of the differences recorded for bacterial concentration was evaluated via comparison between the results of treatment samples for each MOI (BP-B, bacteria and phage—bacterial concentration in the treatment) with the corresponding bacterial concentrations in the control (BC, bacterial concentration in the control) for the different inactivation times, using two-way ANOVA and Bonferroni post hoc tests. A value of *p* < 0.05 was considered to be statistically significant.

## 3. Results

In the present research work, the formulation of a bioactive edible biopolymeric microcapsular wrapping (EBMW) integrating a structurally and functionally stabilized cocktail of two newly isolated lytic phages for *Salmonella enterica* has been proposed, aiming at potential applications in poultry feed for the biocontrol of this pathogen. Two different virulent phages were selected based on their ability to form clear plaques of lysis, which were amplified in a *Salmonella enterica* CCCD-S004 bacterial strain and, to assess their infectious potential for biotechnological applications such as biocontrol of the aforementioned pathogen in live poultry, physicochemical and biological characterization was undertaken, together with the characterization of the EBMW formulation integrating the lytic cocktail.

### 3.1. Bacterial Growth Curve

**Figure 1:**
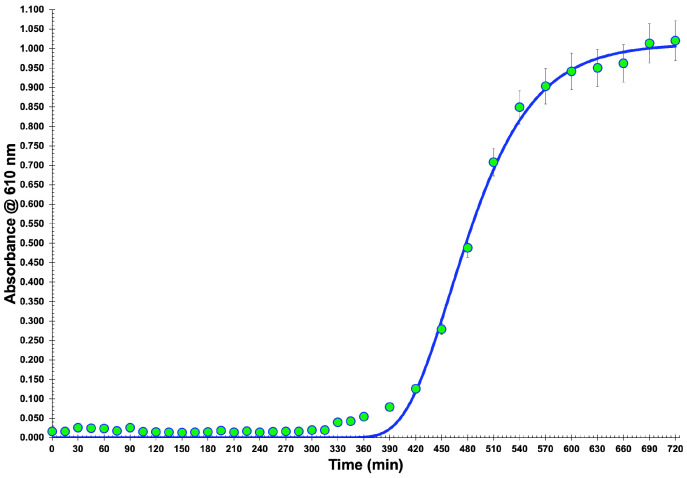
Growth curve of the host bacteria (*Salmonella enterica* CCCD-S004). The nonlinear fitting performed (Gompertz function) allowed estimation of the maximum biomass concentration at t = ∞ of 1.013 × 10^9^ CFU/mL (corresponding to a maximum absorbance of 1.1049), and of the lag period (345 min, ca. 5.75 h). Values represent the mean of three experiments; error bars represent the standard deviation.

For isolating lytic bacteriophage particles, one decided to use a collection bacterium (viz. *Salmonella enterica* CCCD-S004), and therefore the production of a growth curve (Figure 1) was mandatory to observe the growth characteristics of the bacterial cells. For up to 5 h of growth, approximately, the bacterium remained in a latency period and, after this short timeframe, its exponential growth phase began and extended up to 9 h of growth (data not shown). Then, the onset of the stationary phase period could be observed. These results were very important to the subsequent research work, since an active bacterial culture in the exponential phase is necessary for all phases of phage infection, isolation, and amplification. A nonlinear fitting of the Gompertz function was performed on the experimental bacterial growth data, allowing one to estimate the maximum biomass concentration at t = ∞ as 1.013 × 10^9^ CFU/mL (corresponding to a maximum absorbance of 1.1049), and of the lag period as 345 min (ca. 5.75 h).

### 3.2. Phage Virion Morphology via Transmission Electron Microscopy (TEM) Analyses

**Figure 2:**
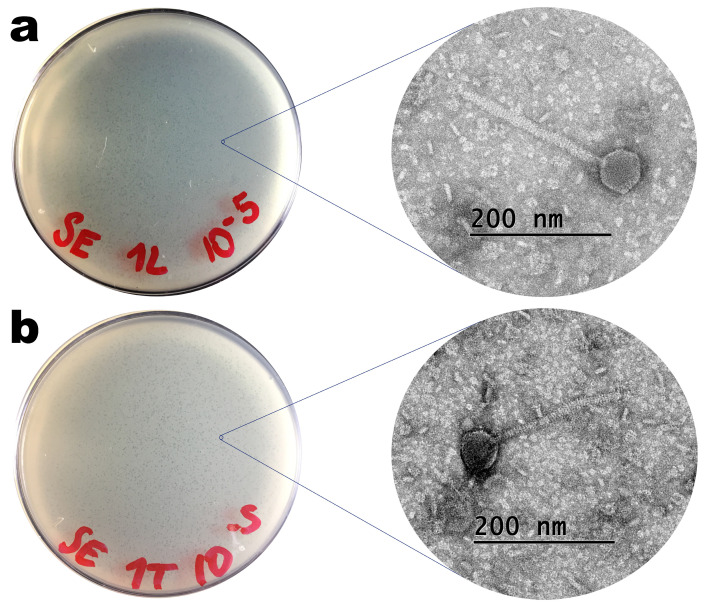
Images of lysis plaques and negative-staining TEM photomicrographs of phages ph001L (**a**) and ph001T (**b**).

Phages ph001L and ph001T, isolated from lake water and soil containing hen faeces in the surroundings of the Veterinary Hospital located within the UNISO Campus (Sorocaba, SP, Brazil), produced clear plaques with very small dimensions on the host (*Salmonella enterica* CCCD-S004) lawn (Figure 2), with absence of secondary halo surrounding them, indicating that these phages probably do not produce depolymerase enzymes [45,46]. TEM photomicrographs of phages ph001L and ph001T can be observed in Figure 2.

Based on the morphological analysis entailed by TEM (Figure 2), both phages displayed siphovirus morphotypes and were putatively identified as belonging to the class Caudoviricetes. Phages ph001L and ph001T displayed perfect icosahedral heads and long, flexible, non-contractile tails; their approximate dimensions are displayed in Table 2.

### 3.3. Phage Particle Extinction Coefficients

**Figure 3:**
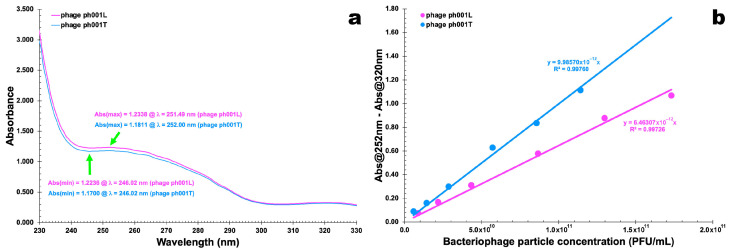
UV-Vis spectral scans of PEG-concentrated phages ph001L and ph001T (**a**) and linear relationships between corrected absorbance and phage particle concentration (**b**).

UV-Vis spectral scans of PEG-concentrated phages ph001L and ph001T (Figure 3a) were used to obtain the wavelength producing the maximum absorption of radiation of both phages, giving rise to the data displayed in Table 3 that was used to determine the phage particle extinction coefficient (Figure 3b).

For the two phages, maximum absorption was observed around 251 nm (phage ph001L) and 252 nm (phage ph001T), and the minimum absorption that was observed around 245 nm was an indication that bacterial cell debris were virtually absent from the preparation with the concomitant presence of a high concentration of phage virions.

Fitting the Beer–Lambert linear relationship to the experimental data displayed in Table 3 (Abs_251(2) nm_ − Abs_320 nm_ = ƒ (phage particle concentration, PFU/mL)), allowed obtaining the molar extinction coefficient of the newly isolated phages as ε_phage ph001L_ = 6.463 × 10^−12^ (PFU/mL)^−1^·cm^−1^ and ε_phage ph001T_ = 9.986 × 10^−12^ (PFU/mL)^−1^·cm^−1^. By subtracting Abs_320nm_, a wavelength where there is little absorption of radiation from phage chromophores, a raw correction for light scattering from phage particles and non-phage particulate contaminants was carried out [23,24,25,26,27,28].

Phage virions are essentially made of structural proteins such as capsid, tail, baseplate, and spike, whose chromophores (essentially the side chains of TRP, TYR, and PHE and disulfide bonds of CYS moieties) exhibit a maximum absorption of radiation around 278–280 nm in the ultraviolet wavelength region. Hence, quenching of radiation owing to protein chromophore absorption and scattering by whole virion particles corrected by the quenching of radiation owing solely to protein chromophores, leads to the hallmark absorbance for a particular virion at a particular concentration [47]. Structural protein chromophores of phage virions have a nearly zero absorption of radiation (completely due to scattering [48]) at 320 nm, which one uses to correct for radiation scattering from virions and other contaminating particulates. Therefore, the structural proteins of the two phages isolated in the research work described herein contributed substantially to their absorption spectrum and were responsible for the wide plateau between 250 and 280 nm in the spectra, with a shallow maximum at 251 nm (phage ph001L) or 252 nm (phage ph001T).

According to previous studies [23,24,25,26,29], the molar extinction coefficient is yet another parameter that allows one to differentiate between isolated phages and, as can be observed in Figure 3b, the two phages are indeed different despite exhibiting the same siphovirus morphotype.

### 3.4. Phage Host Range and Efficiency of Plating (EOP)

Spot testing indicated that phages ph001L and ph001T could form completely cleared zones on 7 of the 19 strains tested (Table 4). Beyond the host isolation strain, phages ph001L and ph001T infected *Salmonella enterica* subsp. Enteritidis ATCC 13076, *Pseudomonas syringae* pv. *Garcae* IBSBF-158, *Escherichia coli* ATCC 25922, *Escherichia coli* ATCC 8739, *Klebsiella pneumoniae* ATCC-13883, and *Klebsiella pneumoniae* NCTC-13439, with moderate efficacies (Table 4).

### 3.5. Phage One-Step Growth (OSG) Analyses

**Figure 4:**
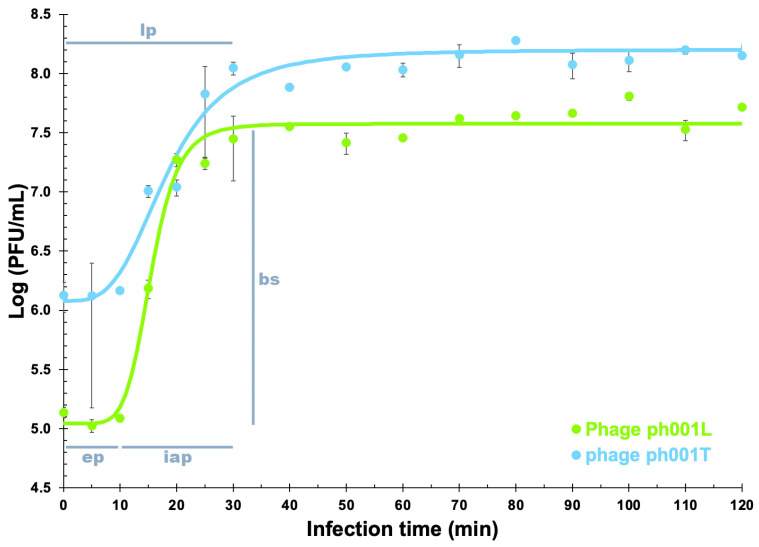
Analysis of the growth curves in a single synchronous cycle of phages (10^5^ PFU/mL) ph001L and ph001T on a late exponential phage culture of the host (*Salmonella enterica* CCCD-S004, 10^8^ CFU/mL) (MOI ≤ 0.001). The fitted 4-PL model to the experimental phage virion growth data, represented by blue and green lines, enabled estimation of phage virion growth features such as eclipse (ep), latent (lp), and host intracellular accumulation (iap) periods, and burst size (bs), as illustrated in Figure 4. All experimental data values represent means of triplicate determinations in three independent experiments. Error bars represent asymmetric standard deviations.

Fitting the experimental one-step phage growth data to the 4-PL model via nonlinear regression enabled one to determine the virion growth features for phages ph001L and ph001T (Figure 4).

The phage growth parameters were determined from the nonlinear fittings performed to the experimental phage growth data. Phage ph001L presented an eclipse period (ep) of 10 min, a latent period (lp) of 25 min, and an intracellular accumulation period (iap) of 15 min, with a virion morphogenesis yield (bs) of 466 virions/host cell, whereas phage ph001T presented an eclipse period (ep) of 10 min, a latent period (lp) of 40 min, and an intracellular accumulation period (iap) of 30 min, with a virion morphogenesis yield (bs) of 132 virions/host cell.

### 3.6. Phage Adsorption Analyses

**Figure 5:**
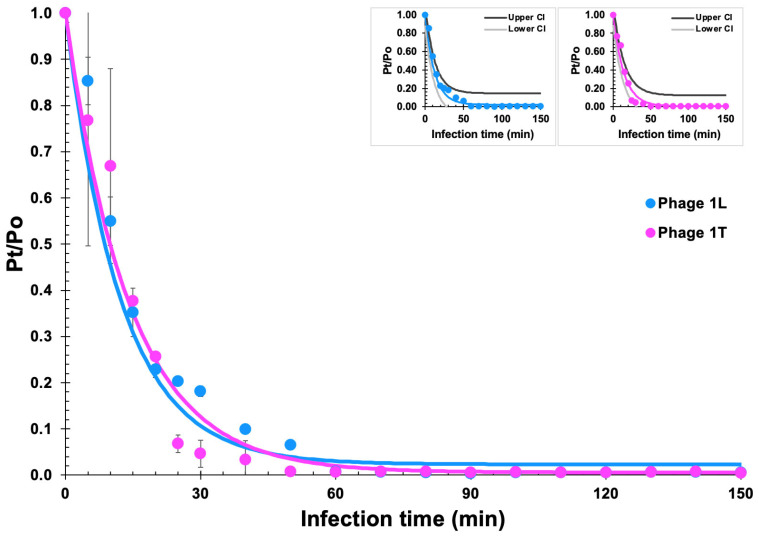
Adsorption curves of phages ph001L and ph001T particles onto their host cells. The fitted adsorption decay model to the experimental phage virion adsorption data, represented by blue and magenta lines, enabled estimation of phage virion adsorption features such as adsorption and desorption rates. All experimental data values represent means of triplicate determinations in three independent experiments. Error bars represent standard deviations.

Phages ph001L and ph001T adsorption assays showed that approximately 90% of the phage particles adsorb to *Salmonella enterica* CCCD-S004 cells after 30 min and 100% adsorbed after 60 min (Figure 5).

Fitting the experimental phage virion adsorption data to the adsorption decay model via nonlinear regression enabled estimation of the phage virion adsorption rates onto their host cells (δ) and desorption rates from virion–bacteria complexes (ϕ): δ_ph001L_ = 8.000 × 10^−10^ CFU^−1^·mL·min^−1^ and φ_ph001L_ = 1.900 × 10^−3^ mL·min^−1^ (*X*_0_ = 1.0 × 10^8^ CFU·mL^−1^; r^2^ (coefficient of determination) = 0.96384); δ_ph001T_ = 7.000 × 10^−10^ CFU^−1^·mL·min^−1^ and φ_ph001T_ = 4.000 × 10^−4^ mL·min^−1^ (*X*_0_ = 1.0 × 10^8^ CFU·mL^−1^; r^2^ (coefficient of determination) = 0.96791).

A statistical test of lack of fit of the adsorption decay model depicted in Section 3.9 was made due to the not-so-small standard deviations of the experimental data points during the first 20 min of the assay, indicating no lack of fit of the mathematical model at a significance level of 0.05 (95% confidence), (phage ph001L: calculated *F*_ratio_ = 0.0868, standard *F*_ratio_ = 2.0147, *p*-value = 0.9999; phage ph001T: calculated *F*_ratio_ = 0.0772, standard *F*_ratio_ = 2.0147, *p*-value = 0.9999). Because “lack of fit” arises from the oscillation of experimental data points around the model fitted, a *p*-value > 0.10 (lack of fit statistically not significant), allows one to conclude that the mathematical model fits (i.e., predicts) the actual response data. Such a conclusion was illustrated via inclusion of small plots in Figure 5, containing upper and lower 95% confidence intervals of the nonlinear fittings performed to the phage adsorption data.

### 3.7. In Vitro Phage–Bacteria Inactivation Assays

**Figure 6:**
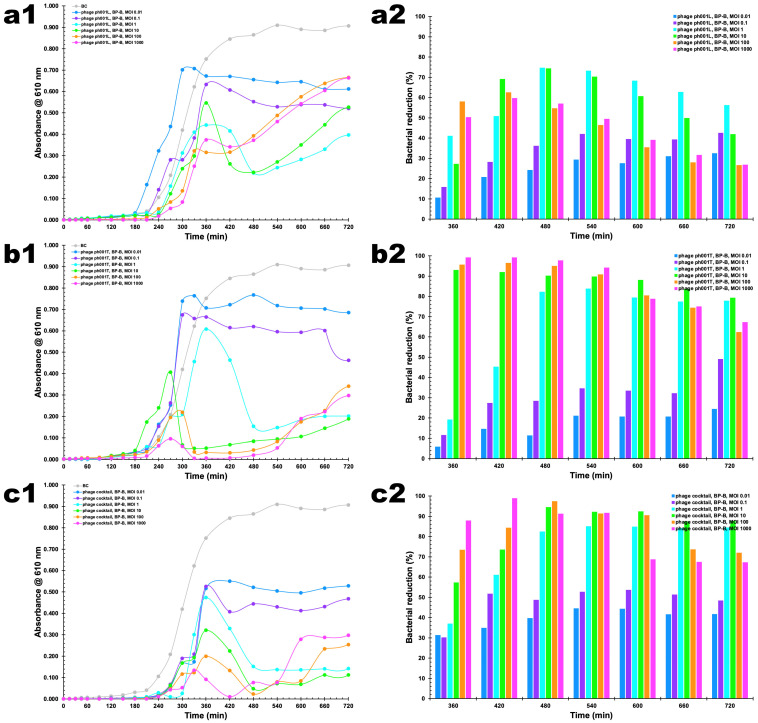
In vitro inactivation of *Salmonella enterica* CCCD-S004 by (**a1**) independent phage ph001L, (**b1**) independent phage ph001T, and by their cocktail (**c1**), at a multiplicity of infection (MOI) of 0.01, 0.1, 1, 10, 100, and 1000, and bacterial reductions (%) produced at all MOI for phages ph001L (**a2**), ph001T (**b2**), and their cocktail (**c2**), during a 12 h treatment timeframe. Bacterial concentration: BC, bacterial control; BP-B, bacteria with phage. Values represent the mean of three independent assays and error bars represent the standard deviation.

The bacterial concentration in the control (BC) increased substantially (ANOVA, *p* < 0.05) during the 12 h of incubation (Figure 6(a1,b1,c1)). When applying the two phages in an independent fashion, phage ph001L did show the worst performance in terms of bacterial reduction (ANOVA, *p* > 0.05) (Figure 6(a1,a2)). At MOI 0.01, phage ph001L managed to reduce the bacterial load by 32.5% after 12 h of incubation with its host. This number increased to 42.5% at MOI 0.1 after 12 h of incubation, and to 56.2% at MOI 1 after 12 h of incubation (Figure 6(a1,a2)). At MOI 1, higher bacterial inactivation was attained after 8 h, viz. 74.6% (ANOVA, *p* < 0.05), with phage ph001L. At MOI 10, the performance of this phage was similar to at MOI 1 (ANOVA, *p* > 0.05). However, at MOI 100, phage ph001L reduced the bacterial load by 58.0% after 6 h of incubation with the host, which was reduced to only 26.6% after 12 h, due to bacterial regrowth (Figure 6(a1,a2)). At MOI values 100 and 1000, the performance of phage ph001L was similar (ANOVA, *p* > 0.05) (Figure 6(a1,a2)). Increasing the MOI from 0.01 → 0.1 → 1 → 100 did significantly increase the inactivation factor after 6 h of incubation (ANOVA, *p* < 0.05), for independently applied phage ph001L (Figure 6), but after 12 h of incubation MOI 100 was not very effective in maintaining bacterial reduction, with the increase in MOI from 0.01 → 0.1 → 1 succeeding in maintaining a significant bacterial reduction (Figure 6(a1,a2)). This scenario was completely changed when phage ph001T was used. After 6 h of incubation with its host, phage ph001T managed to reduce the bacterial load by ca. 93.0% at MOI 10 or 99.2% at MOI 1000, reductions that were maintained at both MOI up to 9 h of incubation (Figure 6(b1,b2)). These bacterial reductions were, however, reduced to not-so-high values after 12 h of incubation, viz. 77.7% (MOI 1), 79.2% (MOI 10), 62.3% (MOI 100), or 67.2% (MOI 1000) (Figure 6(b1,b2)). For phage ph001T, MOI 0.01 was the worst in terms of bacterial reduction performance (Figure 6(b1,b2)), attaining only 24.3% (ANOVA, *p* > 0.05) of bacterial load reduction after 12 h of incubation with the host. Increasing the MOI from 0.01 → 0.1 → 1 → 10 → 100 → 1000 did significantly increase the inactivation factor after 6 h of incubation (ANOVA, *p* < 0.05), for independently applied phage ph001T (Figure 6(b1,b2)). This same trend could be noticed at 7 h, 8 h, and 9 h of incubation of this phage and its host (Figure 6(b1,b2)). As a cocktail, both phages were able to significantly reduce the bacterial load at both MOI 1 and MOI 10, by 84.3% and 87.6%, respectively, after 12 h of incubation with the bacterial host (Figure 6(c1,c2)). At MOI values 1 and 10, the phage cocktail was able to effectively control the bacteria after only 6 h of incubation, maintaining the bacterial reduction at high levels of 84.3% and 87.6%, respectively, up to 12 h of incubation (Figure 6(c1,c2)). The phage cocktail at MOI 10 proved to be the most effective, by significantly (ANOVA, *p* < 0.05) reducing the bacterial load between 6 h and 12 h of incubation with the host (Figure 6(c1,c2)), although MOI 1000 was highly effective between 6 h and 9 h of incubation (Figure 6(c1,c2)). No statistical difference was found for the results produced by the phage cocktail at MOI 1 and MOI 10 after 12 h of Incubation (ANOVA, *p* > 0.05). When phage ph001L was used, a significant bacterial regrowth was observed after 8 h of incubation (ANOVA, *p* < 0.05) for MOI 1, 10, 100, and 1000 (Figure 6(a1,a2)). Regarding phage ph001T, after 8 h of incubation, a slight bacterial regrowth at MOI 1, 10, 100, and 1000 could be observed, until the end of the treatment (Figure 6(b1,b2)). When the two-phage cocktail was used, only a slight (and similar) bacterial regrowth could be observed at MOI 100 and 1000 after 9 h of incubation with the host (ANOVA, *p* > 0.05). Despite this, by the end of the incubation timeframe, the bacterial densities in the different treatments using either independent phages ph001L and ph001T or a cocktail of both phages were significantly lower than that observed for the bacterial control (BC, Figure 6).

### 3.8. Assessment of the Outcome of Abiotic Factors upon Phage Viability

#### 3.8.1. pH Studies

**Figure 7:**
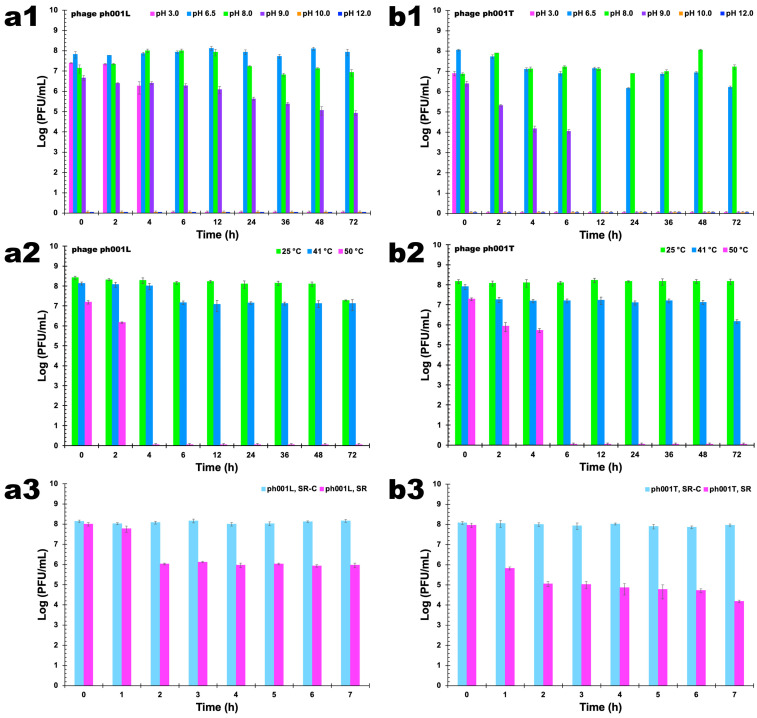
Survival of phages ph001L and ph001T following exposure to different pH values ((**a1**): ph001L; (**b1**): ph001T), different temperature values ((**a2**): ph001L; (**b2**): ph001T), and solar radiation ((**a3**): ph001L; (**b3**): ph001T). All experimental data values represent means of triplicate determinations in three independent experiments. Error bars represent the standard deviation. SR: phage exposed to direct sunlight; SR-C: phage not exposed to sunlight (control).

When different pH values (3.0, 6.5, 8.0, 9.0, 10.0, 12.0) were tested, it was observed that phage ph001L concentration decreased with the decrease in pH; however, the differences among pH values 6.5 and 8.0 were not statistically significant (Figure 7(a1), ANOVA, *p* > 0.05) up to 12 h of incubation, after which phage ph001L viability decreased more at pH 8.0 than at pH 6.5 (Figure 7(a1), ANOVA, *p* < 0.05). However, at pH 3.0, phage ph001L endured during the first 2 h (Figure 7(a1), ANOVA, *p* > 0.05) but lost all its lytic viability (Figure 7(a1), ANOVA, *p* < 0.05) after 4 h. At pH values 6.5 and 8.0, phage ph001L persisted as viable for at least 72 h at 25 °C (Figure 7(a1)). Regarding phage ph001T, 2 h of incubation at pH 3.0 were sufficient to completely inactivate it (Figure 7(b1), ANOVA, *p* < 0.05). After 72 h of incubation at pH 6.5 the abundance of phage ph001T decreased by about two orders of magnitude (Figure 7(b1), ANOVA, *p* < 0.05). Nevertheless, the reduction of lytic viability was more significant at pH 6.5 for this phage than at pH 8.0, after 24 h of incubation (Figure 7(b1), ANOVA, *p* < 0.05). At pH 9.0, phage ph001T lost 1.07 log·PFU/mL after 2 h, 2.35 log·PFU/mL after 6 h, and completely lost its lytic viability after 12 h (Figure 7(b1)). On the other hand, phage ph001L was relatively stable at pH 9.0 up to 12 h of incubation at that pH (Figure 7(a1)), but progressively lost 1.28 log·PFU/mL after 36 h of incubation, 1.59 log·PFU/mL after 48 h, and 1.73 log·PFU/mL after 72 h. At pH values 10 and 12, both phages lost completely their lytic activity immediately after contacting the buffer at those high pH values (Figure 7(a1,b1)).

#### 3.8.2. Temperature Studies

The reduction in the concentration of viable phage ph001L particles was much higher at 50 °C than at 25 °C and 41 °C (Figure 6(a2), ANOVA, *p* < 0.05). A maximum decrease of ca. 1 log·PFU/mL was observed after 72 h when the phage ph001L samples were kept at a temperature of 25 °C, a trend that was also observed at 41 °C (Figure 7(a2)). However, at 50 °C, phage ph001L viability decreased 1 log·PFU/mL after only 2 h of incubation, after which it completely lost its lytic viability (Figure 7(a2), ANOVA, *p* < 0.05). Regarding phage ph001T, it was completely stable at 25 °C (Figure 7(b2), ANOVA, *p* > 0.05), but at 41 °C lost 0.8 log·PFU/mL after 48 h of incubation and ca. 1.8 log·PFU/mL after 72 h (Figure 7(b2), ANOVA, *p* < 0.05). At 50 °C, phage ph001T lost ca. 1.6 log·PFU/mL after only 4 h of incubation, but after this timeframe it ceased to be viable (Figure 7(b2), ANOVA, *p* < 0.05).

#### 3.8.3. Solar radiation Studies

Exposure of phages ph001L and ph001T to direct sunlight for 7 h promoted a decrease of 2.2 log·PFU/mL (phage ph001L, Figure 7(a3), ANOVA, *p* < 0.05) and 3.8 log·PFU/mL (phage ph001T, Figure 7(b3), ANOVA, *p* < 0.05) in the abundance of viable phage virion particles, when compared to the controls (SR-C).

### 3.9. Preparation and Characterization of the Edible Biopolymeric Microcapsular Wrapping (EBMW) Integrating the Bacteriophage Cocktail

**Figure 8:**
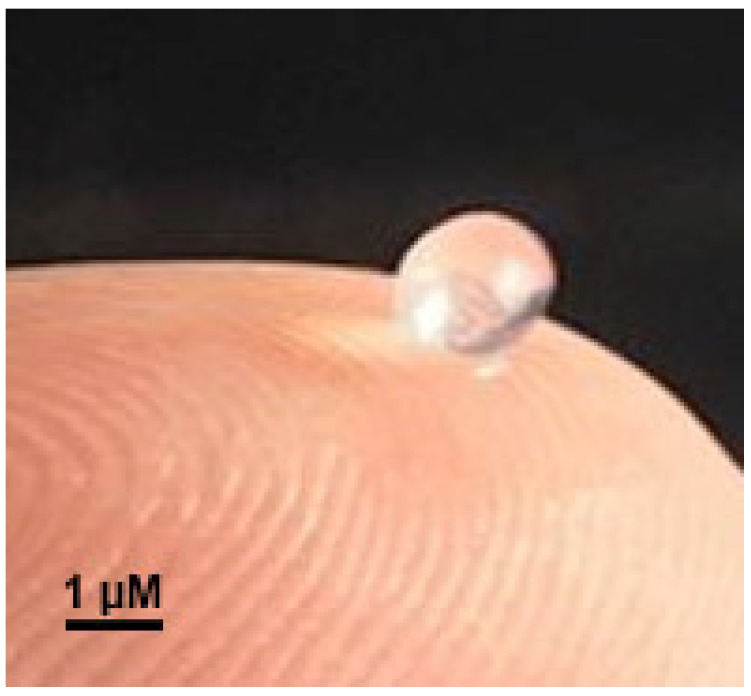
Image of a calcium alginate microcapsular wrapping obtained by ionotropic gelling and integrating a cocktail of two lytic bacteriophages for *Salmonella enterica* CCCD-S004.

The edible biopolymeric microcapsular wrapping was prepared with sodium alginate by inotropic gelling. The technique allowed the preparation of a microcapsular wrapping with translucent and uniform characteristics, with average diameters of 2 µm (Figure 8). Sodium alginate was chosen for the production of the edible microcapsular wrappings for its interesting characteristics linked to biocompatibility, biodegradability, non-toxicity, and gelling capacity [49]. Sodium alginate has been successfully applied in edible films and coatings, aiming at food protection and also as carriers of some food preserving agents (antioxidants and antimicrobials) [50]. Furthermore, other research works have indicated that alginate-based matrices are suitable for phage incorporation and protection [36,37,38,51,52,53].

### 3.10. Assessment of the Lytic Viability of Entrapped Bacteriophage Particles within the EBMW Formulations

**Figure 9:**
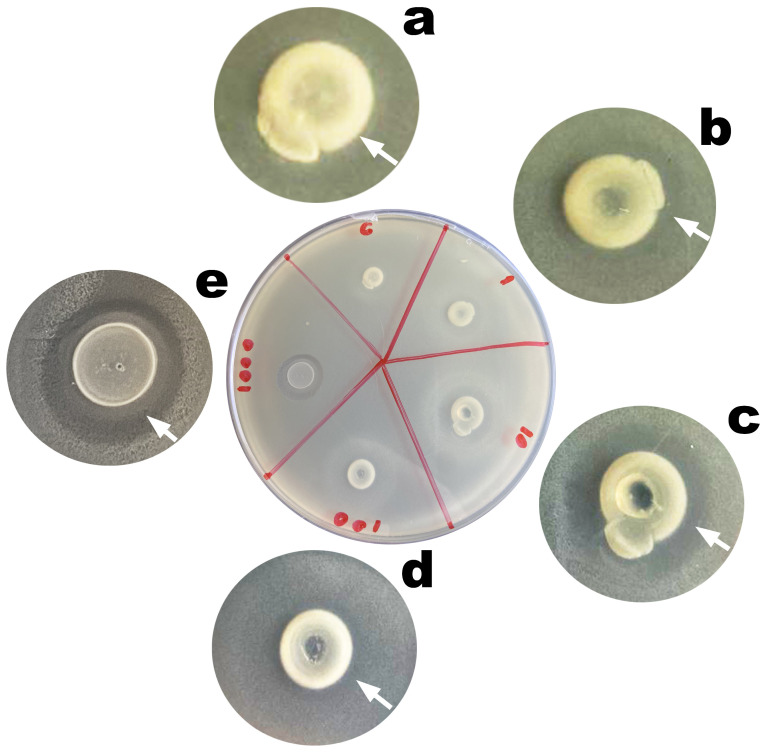
Results from assessment of the lytic viability of entrapped bacteriophage particles within the EBMW formulations. (**a**) EBMW matrix devoid of phage particles, and bioreactive EBMW matrices integrating the phage cocktail at (**b**) MOI 1, (**c**) MOI 10, (**d**) MOI 100, and (**e**) MOI 1000.

Entrapment of the phage virion particles in the chitosan-coated calcium alginate biopolymeric matrix of the EBMW particles promoted structural and functional stabilization of said virions, with maintenance of their lytic viability (Figure 9). Maintenance of the lytic activity of the phage virion particles within the microcapsular wrapping was evaluated since immobilization on different matrices can affect both their viability and availability. The process of obtaining films, coatings, and hydrogels integrating phage particles ends up exposing them to stressful conditions such as mixing, stirring, or drying [54]. Figure 8 displays images of Petri plates containing a lawn of *Salmonella enterica* CCCD-S004 and, on top of it, the microcapsular wrappings (integrating or not phage particles). Lysis zones can be seen in the lawn, surrounding the microcapsular wrappings integrating the phage cocktail at MOI 1 (Figure 9b, inserted arrow), 10 (Figure 9c, inserted arrow), 100 (Figure 9d, inserted arrow), and 1000 (Figure 9e, inserted arrow), indicating maintenance of the lytic activity of the phage particles on the host bacteria upon immobilization within the microcapsular wrapping matrices. No lysis zone could be observed for the control EBMW (Figure 9a).

No lysis zone could be observed in the bacterial lawn surrounding the control microcapsular wrapping matrix (Figure 9a). On the contrary, clear zones of lysis surrounding the EBMW matrices integrating the phage cocktail was most evident for all MOI values tested (Figure 9), with the lysis area increasing in general with increasing MOI, leading to the conclusion that integration of the phage particles within the EBMW matrix formulation did not interfere with the lytic activity of the entrapped phage particles.

**Figure 10:**
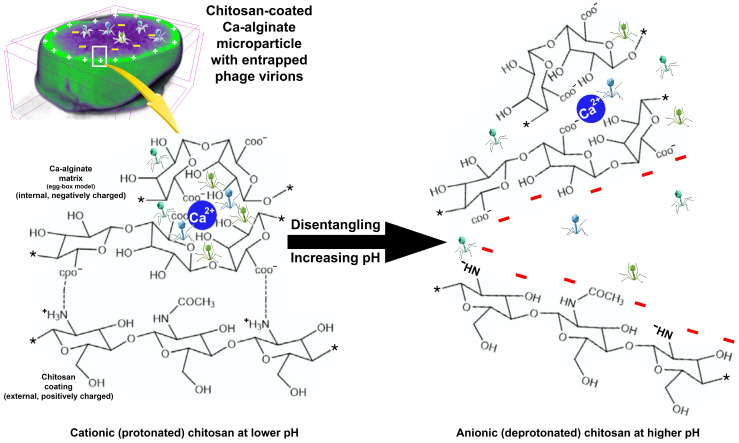
Putative mechanism for the release of entrapped phage virions during incubation with a bacterial lawn of the host, showing the interactions (dotted lines representing hydrogen bonding) between calcium alginate and chitosan. ⋆ denotes repeating polymer monomers.

To try to explain the lysis promoted by the entrapped phage particle cocktail when in contact with a lawn of the host (*Salmonella enterica* CCCD-S004), a putative mechanism was put forward (Figure 10).

Figure 10 displays an illustration for the putative interactions between the Ca alginate matrix and the chitosan coating at different pH values. Chitosan is electrostatically bound to the surface of the Ca alginate matrix at a lower pH (top-agar surface, pH equal to ca. 6). At a higher pH (*Salmonella enterica* lawn surface, pH equal to ca. 9), chitosan becomes deprotonated and acquires a net negative charge, and the repulsion forces acting on the (also negatively charged) Ca alginate matrix prevents surface rebinding. The increase in pH promotes a disentanglement of the two polymers, destructuring the particle and promoting release of the phage virions into the outer medium, where the phage virions can contact and infect the bacterial host cells, promoting their lysis, as can be observed in Figure 9.

**Figure 11:**
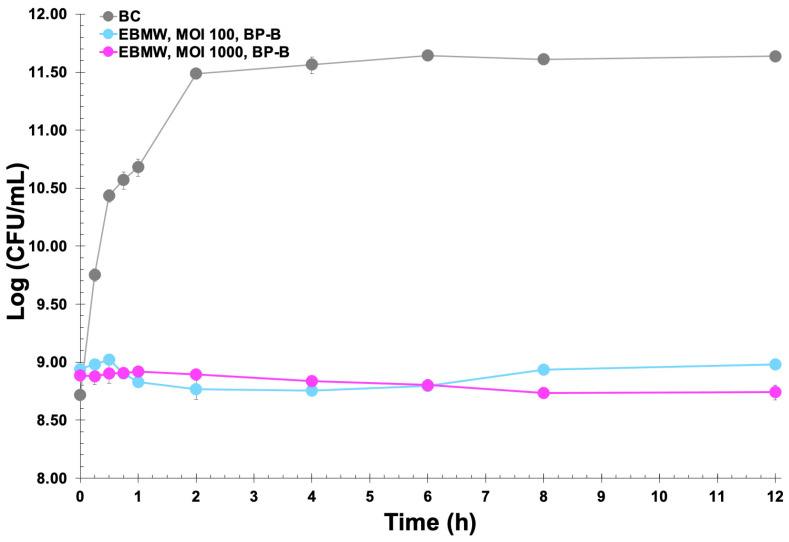
In vitro inactivation of *Salmonella enterica* CCCD-S004 by the entrapped phage cocktail (EBMW) at MOI 100 and MOI 1000, during a 12 h treatment timeframe. Bacterial concentration: BC, bacterial control; BP-B, bacteria with EBMW particles. Values represent the mean of three independent assays and error bars represent the standard deviation.

The EBMW matrices integrating the phage cocktail at MOI 100 and MOI 1000 were also used in microscale bacterial inactivation assays, and the results obtained are displayed in Figure 11.

As can be observed from inspection of the data in Figure 11, the bioactive (lytic) EBMW formulation containing the cocktail of bacteriophages at MOI 1000 was able to promote a reduction in the bacterial load, albeit slight, due most probably to the non-disintegration of the particles within the bacterial suspension and concomitant non-release of all bacteriophage particles. The EBMW with the entrapped phage cocktail at MOI 100 was able to promote a slight decrease of 2.81 log·CFU/mL after 4 h of treatment, which decreased to only 2.68 log·CFU/mL after 8 h and 2.65 log·CFU/mL after 12 h of treatment. On the other hand, the EBMW with the entrapped phage cocktail at MOI 1000 was able to promote a nearly identical decrease in bacterial load after 4 h of treatment, which increased to 2.88 log·CFU/mL after 8 h and endured up to the end of treatment, with a decrease of 2.90 log·CFU/mL after 12 h.

**Figure 12:**
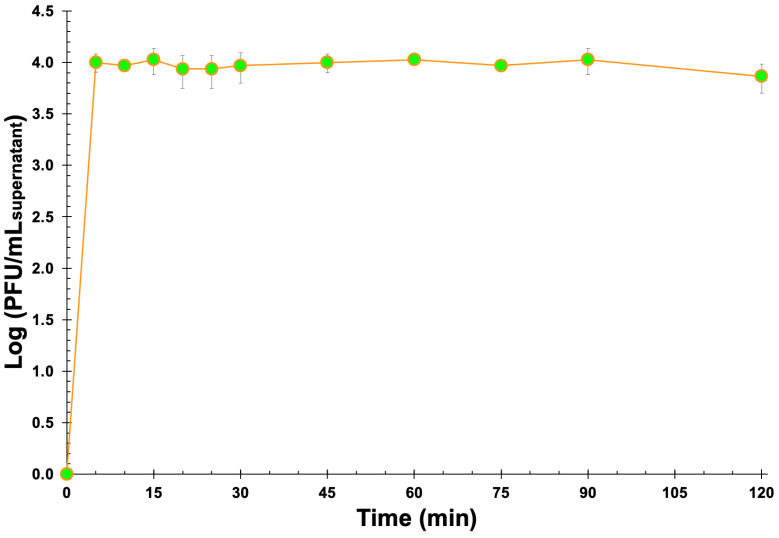
In vitro phage virion release profile from the EBMW particles integrating the phage cocktail at MOI 1000, into plain SM buffer. Values represent the mean of three independent assays and error bars represent the standard deviation.

From the simple experiment that was designed and implemented aiming at evaluating the process of release of the phage virions from the EBMW formulation with entrapped phage particles at MOI 1000, it is clear that the EBMW particles released the virions progressively with time and that they retained their lytic activity (Figure 12).

### 3.11. Fourier Transform InfraRed Spectrometry (FTIR) Analyses

**Figure 13:**
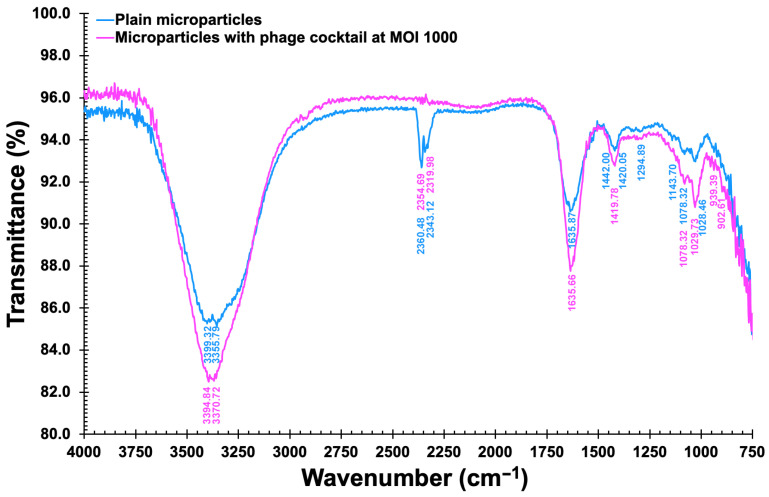
Fourier transform infrared (FTIR) spectra of the chitosan-coated EBMW integrating the phage cocktail at MOI 1000 (pink line) and of the chitosan-coated EBMW devoid of phage particles (blue line).

Figure 13 shows the FTIR spectra of the chitosan-coated EBMW integrating the phage cocktail at MOI 1000 and of the chitosan-coated EBMW devoid of phage particles (control sample).

The FTIR spectra of the chitosan-coated EBMW integrating or not the phage cocktail are very similar (Figure 13), allowing one to conclude that the phage particle did not engage in any type of chemical reaction with the EBMW biopolymeric matrix. The broad peak between 3000 and 3650 cm^−1^ corresponds to the elongation of the OH- groups present in both the alginate polymer chain and residual water molecules [55,56]. The peaks at 1420.05/1419.78 cm^−1^ and 1635.87/1635.66 cm^−1^ may be attributed, respectively, to asymmetrical and symmetrical axial distortions of -COO- groupings, indicative of the existence of carboxylic acid residues in the calcium alginate matrix [55]. Stretching of C=C were encountered at ca. 1636 cm^−1^ for the calcium alginate matrix coated with chitosan, arising most likely from isolated alkenes. The existence of N-acetyl moieties originating from chitosan was ascertained by the peaks appearing at ca. 1635 cm^−1^ (primary amide C=O stretching) and 1295 cm^−1^ (tertiary amide C-N stretching). No characteristic N-H bondings from secondary amides were found in the spectra of the formulations tested. The peaks at ca. 1420 cm^−1^ were attributed to bonding of CH_2_ groups. The small peak at ca. 1144 cm^−1^ was assigned to asymmetrical stretching of -C-O-C- groups whereas the peaks at 1078 cm^−1^ and 1028/1029 cm^−1^ were assigned to stretching vibrations of the -C-O-C- bond of the ether groups from the chitosan coating [57,58,59,60,61]. The peak found at ca. 902 cm^−1^ was attributed to -C-H- groups bonding out of the plane of the sugar rings in chitosan moieties [62], whereas the peak at 1078 cm^−1^ was most likely due to stretching of -C-N- bonds from aliphatic amines [63]. Abnormally, the absorption peak that appeared at 2360 cm^−1^ was most likely due to antisymmetric stretching of CO_2_ molecules from air entrapped inadvertently in the EBMW matrices during their formation [64]. As can be noticed from inspection of Figure 13, the same peaks can be observed (with only minor variations in peak intensity) in the FTIR spectra of plain EBMW microparticles and EBMW microparticles integrating the phage virion cocktail at MOI 1000, strongly suggesting that the chemical features of phage virions were conserved during entrapment within the microcapsular wrappings.

### 3.12. Thermal Characterization of the EBMW Formulations via DSC

**Figure 14:**
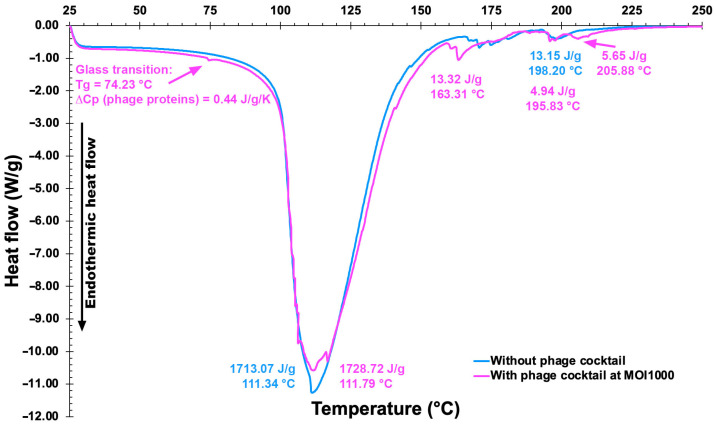
Differential scanning calorimetry thermograms of the EBMW formulation devoid of phage particles (blue line) and of the EBMW formulation integrating the cocktail of phage particles (magenta line).

DSC thermograms of a plain EBMW formulation and of an EBMW formulation integrating the phage virion cocktail at MOI 1000 are displayed in Figure 14.

Very similar thermal events can be perceived for both EBMW particles, with the sample containing the phage virion cocktail displaying a slightly higher melting enthalpy at virtually the same temperature, viz. 111 °C. The peak temperature of heat absorption of the two particle formulations were very close to one another and virtually equal to the mid-point of the calcium alginate melting range, viz. ca. 111.5 °C [65]. The prepared microcapsular wrappings were basically made of chitosan-coated calcium alginate. Thus, one can observe thermal events similar to each other. The first endothermic events (sample and control) are probably related to coating dehydration [66]. The second endothermic events at 163.31 °C (sample) may be due to a depolymerization process. The process of thermal disintegration of (bio)polymers encompass sequential steps of dehydration, depolymerization, and disruption of -C-O- and -C-C- bonds with concomitant production of CO, CO_2_, and H_2_ [67]. The EBMW sample also showed two small endothermic events at higher temperatures, viz. 195.83 °C and 205.88 °C, which might be due to the influence of components in the buffer solution (where the phages are diluted) that increase the conformational stability by electrostatic interactions of the present components [68]. Phages encapsulated in a glassy matrix having a low moisture content, such as the EBMW, may result in better storage stability at low and ambient storage temperatures [69]. In addition, the glass transition temperature (Tg) detected at Tg = 74.23 °C in the EBMW sample containing the phage cocktail might be due to the phage proteins, a value slightly higher than the Tg reported by other researchers [70] for microencapsulated phage against *Salmonella*. According to several researchers [71], protein moieties can be retained dried within a vitreous sugar matrix at temperatures (T) lower by at least 50 °C than Tg, primarily for the reason that at (Tg − T) > 50 °C, protein moieties are sufficiently stagnant with decreased reactivity. Perhaps such vitreous stabilization rationale may also be applied to protein-based entities such as phage virions.

### 3.13. Elemental Profile of the EBMW Formulations Obtained by EDXRF

**Figure 15:**
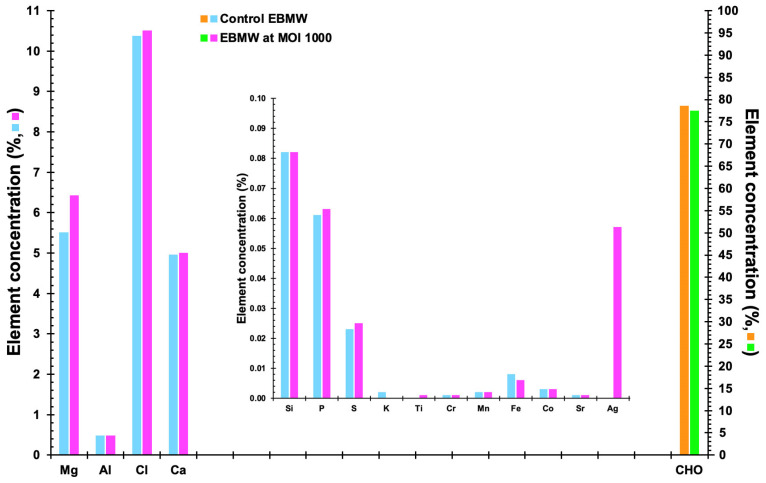
Elemental profiles of EBMW formulations, with and without phage particles.

The elemental profiles of EBMW formulations, with and without phage particles, are displayed in Figure 15. Relatively high concentrations of magnesium (Mg), chlorine (Cl), and calcium (Ca) were found for both formulations, originating probably from the bacteriophage suspension and calcium chloride utilized to prepare the formulations. Al (most likely originating from the sodium alginate itself or chitosan, being probably a contaminant) was detected at ca. 0.4%, and other elements such as phosphorus, sulfur, iron, and silver were detected in very small amounts.

The most common substances were, as expected, carbon, hydrogen, and oxygen, accounting for ca. 79% and 77% of EBMW formulations without and with the phage cocktail, respectively (Figure 15). Calcium alginate, the basis of the edible microcapsular wrapping, is made almost entirely of alginate extracted from seaweed [72], and thus, CHO, calcium, and chlorine, in greater proportions, are derived, probably from alginate. The element calcium (Ca) was also found in higher concentration due to its addition during the inotropic gelation process. The elements phosphorus (P) and magnesium (Mg) also stood out, probably coming from the phage suspensions utilized. However, these elements are not at all considered to be toxic.

### 3.14. XRT Analysis of the EBMW Integrating the Cocktail of Phage Particles

**Figure 16:**
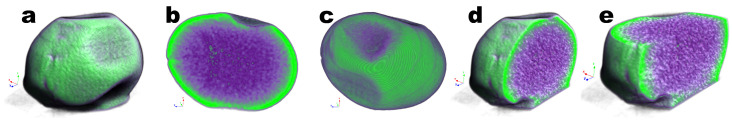
Images obtained by tomographic analyses via X-ray transmission of the EBMW particle loaded with phage particles, being (**a**) front view of a EBMW, (**b**) vertical cut of a EBMW, (**c**) top view of a EBMW, (**d**) frontal cut, and (**e**) EBMW particle with front and top cuts. Three-dimensional image slices were gathered using an operating voltage set at 31 kV and electric current with 661 μA.

The optimized EBMW developed may be considered a natural polymer composite exhibiting a very special porous microstructure which enables the imprisonment of the phage particles. From the tomographic analyses via X-ray transmission performed to an EBMW particle loaded with the phage particle cocktail (Figure 16), a homogeneous surface can be observed.

The chitosan layer coating the calcium alginate matrix is in greater evidence (in green in Figure 16) since, due to its stronger atomic density, it absorbs radiation to a greater extent. On the other hand, the void spaces show up pinpointed in light green within the polymeric network matrix (in purple in Figure 16) in the reconstructed three-dimensional image (Figure 16b,d,e). This closely compares with the information gathered in the FTIR tests (Figure 13), viz. that the virion particles likely did not establish any covalent bondings with the calcium alginate matrix. This realization is clearly significant, meaning that by not establishing permanent bonding with the calcium alginate matrix, the phage particles become readily available and maintain their lytic bioactivity, as was demonstrated before (Figure 9, Figure 11 and Figure 12). A comparative porosity analysis of the EBMW formulation integrating the phage particles can be found in Table 5, resulting from 2D and 3D morphological analyses.

When the properties of a substance, both mechanical and/or physical, differ when determined along a Cartesian coordinate system, it means that such properties are directionally dependent, i.e., are anisotropic. The degree of anisotropy (DA) {$1-\left({\mathrm{Eigenvalue}}_{\min}/{\mathrm{Eigenvalue}}_{\max}\right)\}$ can assume any value in the range 0 (total isotropy)–1 (total anisotropy). The EBMW loaded with phage virions exhibited a DA of 0.63632 (Table 5), and thus can be considered more anisotropic than isotropic. The results obtained for the total porosity of the EBMW (86.58%, Table 5) allow one to conclude that the particles produced were mostly porous in their structure, displaying an open porosity exactly equal to the total porosity (Table 5). Additionally, the mean fractal dimension in the 3D analysis is 2.04, giving a measure of how “intricate” a self-similar figure is and measuring roughly “how many points” lie in a given set. The mean fractal dimension obtained (2.04) has an interesting property in the sense that, as it fills the space of an area, it acts as if it is filling the space of a volume [73].

### 3.15. Morphological Analyses of the EBMW via SEM

**Figure 17:**
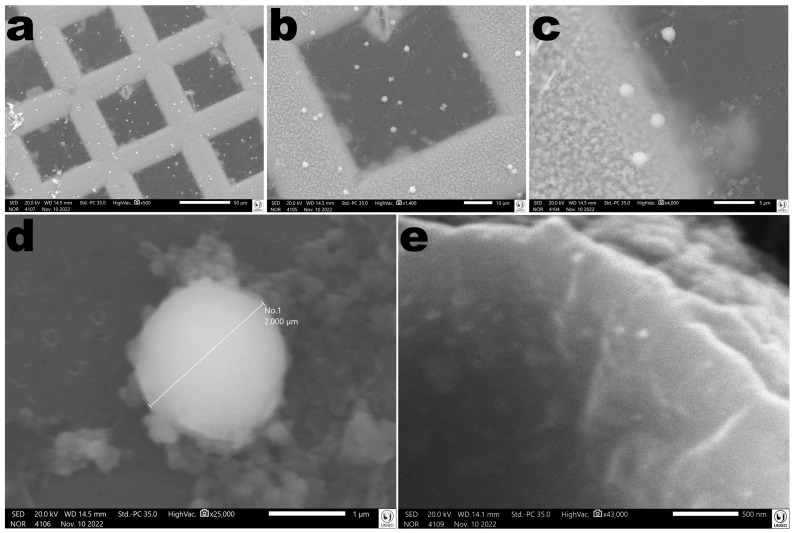
Photomicrographs of the vaginal egg surface at several magnifications ((**a**): ×500; (**b**): ×1400; (**c**): ×4000; (**d**): ×25,000; (**e**): ×43,000). Images obtained by scanning electron microscopy (SEM) confirmed the formation of microcapsular wrappings with homogeneous characteristics. In photomicrograph (**d**) it is possible to observe the mean diameter of the particles produced.

Analyses of a EBMW particle via SEM allowed one to observe a homogeneous surface without any fissures or crevices (Figure 17).

## 4. Discussion

*Salmonella enterica* is known as one of the main microorganisms responsible for poultry contamination. Developing new alternatives to the conventional antibiotic-based antimicrobial control for preventing and/or controlling infections by this pathogen have been quite challenging and a long-time goal within the scientific community, aiming at reducing the development of multi-drug resistant bacteria. In the research effort described herein, the structural and functional stabilization of two newly isolated lytic phages for *S. enterica* (viz. phages ph001L and ph001T, isolated from environmental samples at the Campus of UNISO in Sorocaba, SP, Brazil) within edible biopolymeric microcapsular wrappings (EBMW) has been proposed, aiming at a potential integration in poultry feed as a means to control the aforementioned pathogen. The results obtained in this study provide clear evidence that the use of the two newly isolated phages can reduce the population of pathogenic *S. enterica* cells. The two newly isolated phages produced translucent and tiny plaques on a lawn of the bacterial host, exhibiting diameters of approximately 0.1 mm (Figure 2); were identified as members of the class Caudoviricetes and displayed siphovirus morphotypes (Figure 2) with similar capsid dimensions but with different tail lengths (Table 2); and displayed distinct extinction coefficients (Figure 3b) yet of the same order of magnitude [24,25]. The phage plaques produced by both phages were clear and tiny and did not exhibit a secondary halo in the frontier of the lysis plaque of phage (Figure 2), which is a likely indication that these phages do not produce depolymerase enzymes [74].

In the present study, the host range of the two newly isolated phages was assessed by determining if they were able to form clear plaques of lysis on particular bacteria (meaning that the phages were able to productively infect the bacteria and yield progeny). According to Hyman [75], newly isolated phage particles may also infect different bacterial cells displaying similar receptors on their surface, beyond the species used in their isolation. Besides the isolation strain, phages ph001L and ph001T were able to bind to *Salmonella enterica* subsp. Enteritidis ATCC 13076, *Pseudomonas syringae* pv. *Garcae* IBSBF-158, *Escherichia coli* ATCC 25922, *Escherichia coli* ATCC 8739, *Klebsiella pneumoniae* ATCC-13883, and *Klebsiella pneumoniae* NCTC-13439, and kill them with moderate efficacies (Table 4), yielding progeny virions at relatively high numbers and producing EOP values not so low, as was verified for those bacterial strains, representing 37% of all bacteria tested. According to several authors, while some phages can only infect one or a few bacterial strains, other phages can infect many species or even bacteria from different genera [76,77,78,79], evidence that supports the results obtained in the present research effort for the EOP determinations in the bacterial strains that produced positive spot tests with both phages. Hence, a well-known singularity of bacterial lysis prompted from the inside by phage-derived holins and lysins, a process commonly known as “lysis from within”, can be speculated as a credible reasoning for these bacterial strains exhibiting specific surface receptors recognized by both phages that led to their infection and concomitant killing. Notwithstanding this realization, if practical applications are sought, new (different) lytic phages (isolated from environmental sources) need to be integrated in the cocktail in order to attain a broader lytic spectrum against more strains of *S. enterica*.

A one step of growth was clearly observed for both phages (Figure 4) during the first 10–30 min, which levelled off after this growth. The two phages produced quite large virion progenies (i.e., burst sizes), viz. 466 and 132 virions/host cell, respectively, for phages ph001L and ph001T, suggesting that both phages replicate well in the host with small latencies (25 min for phage ph001L and 40 min for phage ph001T). A number of studies that appear in the specialty literature revealed that using phages producing large virion progenies (morphogenesis yields) within short lytic cycles enhance the efficiency of bacterial control [80,81,82], however large morphogenesis yields are generally followed by considerably longer latencies [83]. The morphogenesis yield of phage ph001L was ca. 4 times larger than that of phage ph001T, but this did not imply a better performance of phage ph001L; on the contrary, inactivation of planktonic host cells in vitro was in general much higher with phage ph001T.

Adsorption of a free phage virion onto a bacterial host cell is the apotheosis of its existence, with the free energy reserve imparted to the virion three-dimensional conformation during its morphogenesis coming into play, with the bacterial surface receptor-specific adsorption of free phage virions dictating their host range [84]. Hence, knowing the dynamics of virion adsorption onto the bacterial host and its concomitant inactivation in in vitro experiments is of utmost importance if use of phage virions is intended to control pathogenic bacteria.

Both phages revealed virtually equal adsorption rates onto the host cells, viz. 8.0 × 10^−10^ CFU^−1^·mL·min^−1^ and 7.0 × 10^−10^ CFU^−1^·mL·min^−1^, for phages ph001L and ph001T, respectively (Figure 5). These results are of the same order of magnitude as the results reported by [85] (lytic phage fSPB adsorption rate on *Salmonella* serovar Paratyphi B, 4.7 × 10^−10^) and [86] (adsorption rate of phage 1 = 2.2 × 10^−10^ mL·min^−1^ and adsorption rate of phage 2 = 1.8 × 10^−10^ mL·min^−1^, onto *Salmonella typhi*), one order of magnitude lower than results reported by [87] (phage PVP-SE1 adsorption rate on *Salmonella enterica* serovar *Enteritidis* strain S1400, 1.00 × 10^−9^ mL·CFU^−1^·PFU^−1^·h^−1^) and [88] (phage SHWT1 adsorption rate on *Salmonella pullorum* SP01, (8.8 ± 0.5) × 10^−9^ mL·min^−1^), and two orders of magnitude lower than the results reported by [89] (phage phi1 adsorption rate onto *Salmonella enterica* = 1.6 × 10^−8^ mL·min^−1^). According to [84], the adsorption constants in some phages are close to the maximally possible values, viz. ca. 1 × 10^–8^ mL·min^−1^, but our results (as the vast majority of the adsorption rates for phages onto their bacterial hosts) were two orders of magnitude lower than such a maximum. Nevertheless, the desorption rate of phage ph001L (1.900 × 10^−3^ mL·min^−1^) was much larger than that of phage ph001T (4.000 × 10^−4^ mL·min^−1^), implying that fewer phage ph001L virions endured adsorbed to the host cells. Because adsorption of phage virions onto specific receptors on the host cell followed by the virion genome translocation into the host cytoplasm is required for its effective infection and concomitant virion morphogenesis [23,24,90], the much larger desorption rate for phage ph001L may have been accountable for the smaller bacterial inactivation rates promoted by this phage at all MOI studied (Figure 6). The adsorption profile showed that after 30 min ca. 90% of phages ph001L and ph001T particles were adsorbed onto the host cells (Figure 5), whereas after 60 min ca. 100% of the phage particles were adsorbed onto the host cells. As a consequence of the much higher desorption rate, phage ph001L was not able to promote a significant decrease in bacterial concentration at all MOI studied, compared with the non-treated BC (Figure 6(a1,a2)). During the first 6 h of incubation of phage ph001L in the presence of its host, the bacterial concentration was only slightly reduced at all MOI, compared with that of the bacterial control (Figure 6(a1)). On the other hand, phage ph001T, by having a much lower desorption rate, succeeded in promoting significant bacterial reductions, especially at MOI 1, 10, 100, and 1000 (Figure 6(b1,b2)). When incubating the phage cocktail integrating both phages ph001L and ph001T with its host, significant bacterial reductions were observed especially at MOI 1, 10, 100, and 1000 (Figure 6(c1,c2)). A number of studies found in the specialty literature revealed that the decrease in bacterial cell numbers is either stronger or faster at higher MOI [25,91,92,93]. In this work, for either phage, increasing MOI from 0.01 → 0.1 did not significantly increase the efficacy of phage-based treatment (Figure 6(a1,b1,c1)) but increasing MOI from 1 → 10 was much more effective than when increasing MOI from 10 → 100 or from 100 → 1000.

When tested against abiotic factors such as pH, temperature, and solar radiation, phage ph001L concentration decreased with decreasing pH; however, the differences among pH values 6.5 and 8.0 were not statistically significant up to 12 h of incubation, after which phage ph001L viability decreased more at pH 8.0 than at pH 6.5. However, at pH 3.0, phage ph001L endured during the first 2 h but lost all its lytic viability after 4 h. At pH values 6.5 and 8.0, phage ph001L persisted as viable for at least 72 h at 25 °C (Figure 7(a1)). Regarding phage ph001T, 2 h of incubation at pH 3.0 were sufficient to completely inactivate it (Figure 7(b1)). After 72 h of incubation at pH 6.5 the abundance of phage ph001T decreased about two orders of magnitude (Figure 7(b1)). Nevertheless, the reduction of lytic viability was more significant at pH 6.5 for this phage than at pH 8.0, after 24 h of incubation (Figure 7(b1)). At pH 9.0, phage ph001T was not very stable, fully losing its lytic viability after 12 h (Figure 7(b1)). On the other hand, phage ph001L was relatively stable at pH 9.0 up to 12 h of incubation at that pH (Figure 7(a1)), but progressively lost up to 1.73 log·PFU/mL after 72 h of incubation at that pH. At pH values 10 and 12, both phages lost completely their lytic activity immediately after contacting the buffer at those high pH values (Figure 7(a1,b1)). The reduction in the concentration of viable phage ph001L particles was much higher at 50 °C than at 25 °C and 41 °C (Figure 7(a2)). A maximum decrease of ca. 1 log·PFU/mL was observed after 72 h when the phage ph001L samples were kept at a temperature of 25 °C, a trend that was also observed at 41 °C (Figure 7(a2)). However, at 50 °C, phage ph001L viability decreased by 1 log·PFU/mL after only 2 h of incubation, after which it completely lost its lytic viability (Figure 7(a2)). Regarding phage ph001T, it was completely stable at 25 °C (Figure 7(b2)), but at 41 °C lost 0.8 log·PFU/mL after 48 h of incubation and ca. 1.8 log·PFU/mL after 72 h (Figure 7(b2)). At 50 °C, phage ph001T lost ca. 1.6 log·PFU/mL after only 4 h of incubation, but after this timeframe it ceased to be viable (Figure 7(b2)). When phage ph001L was exposed to solar radiation, the abundance of phage particles decreased by 2.2 log·PFU/mL (Figure 7(a3)) after 7 h of exposure, when compared with the phage control (SR-C). The decrease in phage abundance was 2.0 log·PFU/mL (Figure 7(a3)). When phage ph001T was exposed to solar radiation, the abundance of phage particles decreased by 3.8 log·PFU/mL (Figure 7(b3)) after 7 h of exposure, when compared with the phage control (SR-C). The decrease in phage abundance was 3.8 log·PFU/mL (Figure 7(b3)).

*Salmonella enterica* was not effectively inactivated by the cocktail of both isolated phages at MOI 0.01 and 0.1 (Figure 6(c1)), and all remaining MOI tested failed to fully prevent bacterial regrowth (Figure 6(c1)). A number of studies found in the specialty literature state that, because of the gargantuan variability in bacterial cell surface receptors recognized by phage virions, regrowth of bacteria after treatment with phages can be virtually circumvented by using a cocktail composed of a variety of lytic phages displaying different adsorption processes [28,29,80,94,95,96,97].

One of the many challenges faced when performing bacterial biocontrol studies using phage virions resides in proving its feasibility in real-world situations [24,28,29] and, therefore, integration of the phage virions as a cocktail within the chitosan-coated calcium alginate matrix formulation (EBMW) was performed aiming at proving its suitability for inclusion in poultry feed. The process of entrapment of the phage particles via ionotropic gelation did not impact negatively in the lytic viability of the imprisoned phage virions (Figure 9), a conclusion backed by the formation of clear zones of lysis surrounding the EBMW formulations containing phages at different MOI on the *S. enterica* lawn (Figure 9b–e). This was a clear indication that the phage virions imprisoned within the calcium alginate matrix retained their lytic viability [26,27,38,98], with the biopolymeric matrix providing suitable diffusion of the phage particles towards their host cells.

Hence, new in vitro phage–bacteria inactivation assays were performed in microscale, using the EBMW particles containing entrapped phage virion cocktails at MOI 100 and MOI 1000. These experiments, together with the putative mechanism developed (Figure 10), indeed gave a better understanding of how EBMWs with entrapped phages will work in the avian digestive tract. In bacterial suspension in TSB (pH ≈ 7), the chitosan-coated alginate microparticles remain cohesive and firm since the cationic (protonated) chitosan coating stays firmly electrostatically attracted to the Ca alginate matrix (negatively charged). This impairs the full release of phage particles into the suspension, with concomitant low levels of bacterial inactivation, as can be observed in Figure 11. In the avian digestive tract, where pH changes from 2.5–3.5 in the gizzard to 5–6 in the duodenum, 6.5–7.0 in the jejunum, 7.0–7.5 in the ileum, and 8.0 in the cecum/colon, this effect is anticipated to be much more pronounced since separation of the chitosan layer due to its deprotonation at higher pH values leads to disintegration of the EBMW particles. Hence, according to the putative phage release mechanism deployed (Figure 10), release of phage virions from the EBMW formulations is expected to occur easily.

The FTIR spectra of the chitosan-coated EBMW integrating or not the phage cocktail were very similar (Figure 13), allowing one to conclude that the phage virions did not engage in any types of chemical reactions with the EBMW biopolymeric matrix, which suggests that the chemical aspect of the phage virions was fully preserved during incorporation into the biopolysaccharide microcapsular wrapping, a very important conclusion, because if the phages were involved in any type of bonding with the biopolymeric matrix, that would prevent their release.

The prepared microcapsular wrappings were basically made of chitosan-coated calcium alginate, hence similar (major) thermal events were observed for both the EBMW formulation devoid of phage particles and the EBMW formulation integrating the cocktail of phage particles (Figure 14), with melting enthalpies of the same order of magnitude, with the EBMW formulation integrating the phage cocktail absorbing a slightly higher amount of energy (viz. 1728.72 J/g) at virtually the same melting point (Figure 10), viz. 111.79 °C, than the EBMW formulation devoid of phage particles (1713.07 J/g at 111.34 °C). Integration of the phage cocktail in the EBMW formulation promoted a slight increase in the melting enthalpy and displaced slightly the peak temperature of the major thermal event (Figure 14).

The results from the thermal characterization of the EBMW particles are linked to the stability of the particles, a crucial parameter for their successful incorporation into poultry feeding. The thermal stability of a biopolymer can be defined as its ability to withstand the action of heat while maintaining its properties (such as toughness or elasticity) at a given temperature and as can be observed from inspection of Figure 14, the melting temperature peak was around 111 °C. Since both phages retained most of their lytic activity at 41 °C (Figure 7), the thermal analysis confirmed the stability of the EBMW microparticles at temperatures up to 41 °C at least, since the biopolymeric matrix melts down at a much higher temperature (Figure 14).

As expected, the EBMW formulations were composed almost entirely of carbon, hydrogen, and oxygen (Figure 15), arising from the calcium alginate, chitosan, and also from the virions integrated into the antibacterial EBMW formulation.

Analysis of EBMW containing the cocktail of lytic virions at MOI 1000 by XRT (Figure 16) closely compared with the FTIR analysis (Figure 13) and denoted a lack of establishment of covalent bonding between the phage virions and the calcium alginate matrix, thus making the phage virions readily available with maintenance of their lytic bioactivity, as was demonstrated before (Figure 9, Figure 11 and Figure 12). The EBMW formulation produced is essentially anisotropic, with a highly porous (viz. 86.58%) structure and a high mean fractal dimension in the 3D analysis (Figure 16).

Additionally, morphology analysis of a EBMW particle via SEM allowed one to observe a homogeneous rugged surface (photomicrographs c, d, and e in Figure 17), devoid of any fissures or crevices whatsoever. From observation of the photomicrographs in Figure 17, a highly uniform and compact matrix structure can be clearly observed, in clear agreement with results from X-ray tomography (Figure 16). These observations are very significant, since the phage particles were uniformly dispersed within the biopolymeric matrix, and the three-dimensionally reconstructed digital slices in Figure 16 allow the clear observation of the compactness of the formulation, with plenty of hydrophilic pores without air pockets (hydrophobic in nature, which could negatively impact phage viability).

The results described herein clearly suggest that the cocktail produced with the two newly isolated lytic phages, ph001L and ph001T, have the potential to be an effective surrogate to antibiotics in controlling *Salmonella enterica.* Yet, both phages could not fully restrain bacterial regrowth in vitro, neither separately nor as a cocktail. Hence, selecting more lytic phages for phage–bacteria inactivation assays should consider not only their efficacy but also their potential for developing phage-resistant bacterial mutants, which should circumvent and virtually obliterate the downside of bacterial-acquired resistance to phages.

## 5. Conclusions

The results of the present work suggest that phage treatment using phages ph001L and ph001T can be an effective alternative to control *Salmonella enterica.* However, both phages were not able to fully prevent bacterial regrowth, although the bacterial reduction levels were quite high when both phages were used as a cocktail, especially at MOI 1 and 10. It was also demonstrated that microencapsulation within a biopolymeric formulation is a viable method for fully stabilizing the virion particles, both from structural and functional points of view, if integration within poultry feed is sought as a means of controlling *Salmonella enterica* in poultry.

## Figures and Tables

**Table 1:** Final (qualitative and quantitative) compositions of all edible biopolymeric microcapsular wrapping (EBMW) formulations encompassing calcium alginate biopolymeric matrices with entrapped phage particles.

Component		EBMW Formulation				
		EBMW 1: No Phages	EBMW 2: MOI 1	EBMW 3: MOI 10	EBMW 4: MOI 100	EBMW 5: MOI 1000
Phage ph001L (amount of virions)		-	8.63 × 10^7^	5.18 × 10^8^	5.18 × 10^9^	9.94 × 10^9^
Phage ph001T (amount of virions)		-	5.70 × 10^7^	5.70 × 10^8^	5.70 × 10^9^	1.09 × 10^10^
Dispersion A	Phage buffer (mL)	5	5	5	5	5
	Gelatin (mg)	50	50	50	50	50
	Phage cocktail (virions)	-	1.43 × 10^8^	1.09 × 10^9^	1.09 × 10^10^	2.09 × 10^10^
	Sodium alginate at 2% (*w*/*w*) (mL)	20	20	20	20	20
Dispersion B	Calcium chloride 2.65 mol dm^−3^ with chitosan at 0.3% (*w*/*w*) (mL)	10	10	10	10	10

**Table 2:** Approximate dimensions of the two newly isolated bacteriophages.

Dimension	Phage ph001L	Phage ph001T
Head length (nm)	56.1 ± 1.7	52.6 ± 1.7
Head width (nm)	56.9 ± 2.6	57.1 ± 0.0
Tail length (nm)	217.9 ± 1.8	185.2 ± 2.7
Tail thickness (nm)	16.8 ± 1.4	12.8 ± 1.9

**Table 3:** Data used to determine the (whole) phage particle extinction coefficients.

Phage Suspension Volume (μL)	Dilution Volume (μL)	Virion	Number of Phage Virions Withdrawn from Suspension	Virion Particle Concentration (PFU/mL)	Absorbance at 251 (or 252) nm	Absorbance at 320 nm	Absorbance at 251 (or 252) nm—Absorbance at 320 nm
10	2000	ph001L	1.73 × 10^10^	8.65 × 10^9^	0.106	0.032	0.075
		ph001T	1.14 × 10^10^	5.70 × 10^9^	0.135	0.045	0.090
25	2000	ph001L	4.33 × 10^10^	2.16 × 10^10^	0.250	0.082	0.168
		ph001T	2.85 × 10^10^	1.43 × 10^10^	0.226	0.065	0.161
50	2000	ph001L	8.65 × 10^10^	4.33 × 10^10^	0.451	0.141	0.310
		ph001T	5.70 × 10^10^	2.85 × 10^10^	0.429	0.130	0.299
100	2000	ph001L	1.73 × 10^11^	8.65 × 10^10^	0.841	0.263	0.577
		ph001T	1.14 × 10^11^	5.70 × 10^10^	0.905	0.277	0.628
150	2000	ph001L	2.60 × 10^11^	1.30 × 10^11^	1.272	0.394	0.878
		ph001T	1.71 × 10^11^	8.55 × 10^10^	1.210	0.374	0.836
200	2000	ph001L	3.46 × 10^11^	1.73 × 10^11^	1.544	0.476	1.068
		ph001T	2.28 × 10^11^	1.14 × 10^11^	1.610	0.495	1.115

**Table 4:** Host range of phages ph001L and ph001T, evaluated on 19 collection strains. (+): clear zone of lysis; (−): absence of lysis.

Bacterial Strains	Source	Phage ph001L				Phage ph001T			
		Spot Test	Titre in Target Bacteria (PFU/mL)	EOP (%)	Score	Spot Test	Titre in Target Bacteria (PFU/mL)	EOP (%)	Score
*Salmonella enterica* CCCD-S004	Collection, CEFAR	+	1.42 × 10^12^	100 (host)	High	+	1.82 × 10^12^	100 (host)	High
*Salmonella enterica* subsp. Enteritidis ATCC 13076	Collection, ATCC	+	6.87 × 10^11^	48.4	Moderate	+	6.06 × 10^11^	33.3	Moderate
*Pseudomonas syringae* pv. *Garcae* IBSBF-158	Collection, IBSBF	+	2.10 × 10^11^	14.8	Moderate	+	3.26 × 10^11^	17.9	Moderate
*Escherichia coli* ATCC 25922	Collection, ATCC	+	2.18 × 10^11^	15.4	Moderate	+	1.78 × 10^11^	9.8	Moderate
*Escherichia coli* ATCC 8739	Collection, ATCC	+	1.89 × 10^11^	13.3	Moderate	+	2.25 × 10^11^	12.4	Moderate
*Klebsiella pneumoniae* ATCC-13883	Collection, ATCC	+	1.04 × 10^11^	7.3	Moderate	+	4.12 × 10^11^	22.7	Moderate
*Klebsiella pneumoniae* NCTC-13439	Collection, NCTC	+	9.41 × 10^10^	6.6	Moderate	+	3.29 × 10^11^	18.1	Moderate
*Aeromonas hydrophyla* ATCC-7966	Collection, ATCC	−	−	−	−	−	−	−	−
*Enterococcus faecalis* ATCC-29212	Collection, ATCC	−	−	−	−	−	−	−	−
*Proteus mirabilis* ATCC 25933	Collection, ATCC	−	−	−	−	−	−	−	−
*Pseudomonas aeruginosa* ATCC 27853	Collection, ATCC	−	−	−	−	−	−	−	−
*Pseudomonas aeruginosa* ATCC 9027	Collection, ATCC	−	−	−	−	−	−	−	−
*Staphylococcus aureus* ATCC 25923	Collection, ATCC	−	−	−	−	−	−	−	−
*Staphylococcus aureus* ATCC 6538	Collection, ATCC	−	−	−	−	−	−	−	−
*Salmonella enterica* subsp. *Enterica* serovar Typhimurium ATCC 14028	Collection, ATCC	−	−	−	−	−	−	−	−
*Pseudomonas aeruginosa* CCCD-P004	Collection, CEFAR	−	−	−	−	−	−	−	−
*Bacillus cereus* ATCC 14579	Collection, CEFAR	−	−	−	−	−	−	−	−
*Proteus mirabilis* CCCD-P001	Collection, CEFAR	−	−	−	−	−	−	−	−
*Salmonella thyphimurium* ATCC 13311	Collection, ATCC	−	−	−	−	−	−	−	−

**Table 5:** Bi- and three-dimensional morphological parameters of the EBMW containing the cocktail of phage virions at MOI 1000.

Parameter	EBMW Entrapping the Cocktail of Virion Particles at MOI 1000	
	Bi-Dimensional (2D) Morphological Analysis	Three-Dimensional (3D) Morphological Analysis
Number of layers	-	101.0
Pixel size (µm)	-	6.70
Total VOI (volume of interest), TV (µm^3^)	6.45 × 10^9^	6.45 × 10^9^
Object volume, Obj.V (µm^3^)	8.67 × 10^8^	8.66 × 10^8^
Percent object volume, Obj.V/TV (%)	13.44	13.42
Total VOI surface, TS (µm^2^)	2.74 × 10^7^	2.74 × 10^7^
Object surface, Obj.S (µm^2^)	6.13 × 10^6^	5.41 × 10^6^
Total intersection surface, i.S (µm^2^)	0	1.92 × 10^6^
Object surface/volume ratio, Obj.S/Obj.V (µm^−1^)	7.07 × 10^−3^	6.25 × 10^−3^
Mean number of objects per slice, Obj.N	1.15	-
Average object area per slice, Av.Obj.Ar (µm^2^)	1.20 × 10^6^	-
Average moment of inertia (x), Av.MMI (x) (µm^4^)	1.79 × 10^11^	1.51 × 10^14^
Average moment of inertia (y), Av.MMI (y) (µm^4^)	1.00 × 10^11^	9.83 × 10^13^
Average moment of inertia (z), Av.MMI (z) (µm^4^)	-	1.90 × 10^14^
Mean eccentricity, Ecc	0.69	-
Cross-sectional thickness, Cs.Th (µm)	573.70	-
Object surface density, Obj.S/TV (µm^−1^)	-	8.38 × 10^−4^
Mean surface convexity index, SCv.I (µm^−1^)	2.17 × 10^−4^	3.14 × 10^−3^
Degree of anisotropy, DA	-	2.75 (0.64)
Eigenvalue 1	-	2.86 × 10^−2^
Eigenvalue 2	-	4.32 × 10^−2^
Eigenvalue 3	-	7.88 × 10^−2^
Number of closed pores, Po.N (cl)	-	0
Volume of closed pores, Po.V (cl) (µm^3^)	-	0
Surface of closed pores, Po.S (cl) (µm^2^)	-	0
Closed porosity (percent), Po (cl) (%)	9.47 × 10^−4^	0
Mean fractal dimension, FD	1.02	2.04
Volume of open pore space, Po.V (op) (µm^3^)	-	5.58 × 10^9^
Open porosity (percent), Po (op) (%)	-	86.58
Total volume of pore space, Po.V (tot) (µm^3^)	-	5.58 × 10^9^
Total porosity (percent), Po(tot) (%)	-	86.58
Euler number, Eu.N	-	1
Connectivity, Conn	-	3
Connectivity density, Conn.Dn (µm^−3^)	-	0

## Data Availability

Data will be made available upon request.
